# Shape-Controlled Synthesis of Platinum-Based Nanocrystals and Their Electrocatalytic Applications in Fuel Cells

**DOI:** 10.1007/s40820-023-01060-2

**Published:** 2023-03-31

**Authors:** Can Li, N. Clament Sagaya Selvam, Jiye Fang

**Affiliations:** Department of Chemistry, State University of New York at Binghamton, Binghamton, NY USA

**Keywords:** Shape-control, Colloidal synthesis, Pt-based nanocrystals, Electrochemical catalysis

## Abstract

To achieve environmentally benign energy conversion with the carbon neutrality target via electrochemical reactions, the innovation of electrocatalysts plays a vital role in the enablement of renewable resources. Nowadays, Pt-based nanocrystals (NCs) have been identified as one class of the most promising candidates to efficiently catalyze both the half-reactions in hydrogen- and hydrocarbon-based fuel cells. Here, we thoroughly discuss the key achievement in developing shape-controlled Pt and Pt-based NCs, and their electrochemical applications in fuel cells. We begin with a mechanistic discussion on how the morphology can be precisely controlled in a colloidal system, followed by highlighting the advanced development of shape-controlled Pt, Pt-alloy, Pt-based core@shell NCs, Pt-based nanocages, and Pt-based intermetallic compounds. We then select some case studies on models of typical reactions (oxygen reduction reaction at the cathode and small molecular oxidation reaction at the anode) that are enhanced by the shape-controlled Pt-based nanocatalysts. Finally, we provide an outlook on the potential challenges of shape-controlled nanocatalysts and envision their perspective with suggestions.

## Highlights

- Synthetic mechanisms of shape-controlled Pt-based alloy and intermetallic nanocrystals are outlined, and strategies for the design and development of morphology-controlled Pt-based nanostructures are discussed.
- Advanced characterizations and electrochemical applications of these Pt-based nanocatalysts are highlighted.
- Advances and perspectives in designing outperformance and the long-durability of Pt-based nanocatalysts with shape control in this electrochemical field are proposed.

## Introduction

Platinum (Pt) metal, one of the six platinum-group metals as scarce elements in the earth’s crust, has received wild recognition for its extensive applications, especially in thermal and electrochemical catalysis [1–3]. Remarkably, at the nanoscale with precisely controlled morphology, size, composition, structure, and crystal phase, Pt-based nanocrystals (NCs) demonstrate intriguing electrochemical performance [4–6]. By incorporating second or multiple metals into the Pt lattice, alloyed catalysts with enhanced performance could be further created. Unlike random alloy NCs with atomically disordered arrangements, intermetallic catalysts with atomically ordered structures usually demonstrate unique electrocatalytic properties. The disordered alloy NCs can be rationally converted to ordered intermetallic NCs under specific conditions or vice versa. For most Pt-based systems, random alloy NCs can be directly produced from a hot colloidal synthesis, and a post-annealing may convert them into an intermetallic phase [7–11]. However, there are some “unusual” systems in which the atomically ordered phase is stable. For example, a colloidal synthesis at 240 ℃ yielded atomically ordered Pt_3_Sn nanocubes (NCbs), and further thermal treatment resulted in a transition to disordered structures with different performances toward oxygen reduction reaction (ORR) [12].

For Pt-based NCs, their morphology may play a crucial role associated with their atomic ordering/disordering phase in the expressed physicochemical properties. A classic example can be found in the Pt-Ni system [13–16]. It was proven that the (111)-facet of Pt_3_Ni single crystal exhibits superior electrochemical activity toward ORR in acidic media compared to its (100)- and (110)-facets [13]. Notably, by minimizing their sizes to the nanoscale (*e.g.*, ~ 10 nm), Pt–Ni octahedral NCs terminated with the exclusive (111)-facets still show ~ fivefold higher in ORR than that of NCbs with (100)-facets with a similar size [17], indicating that the fascinating performance of NCs highly depends on their morphology. Inspired by the incredible results, tremendous efforts have been devoted to the design and development of various synthetic approaches to control the morphology of Pt-based NCs over the past decades [18–22]. In addition to their impact of morphology and structure on the physicochemical properties, other factors such as the strong metal-support interaction (SMSI) could affect the catalytic performance as well [23–26]. A classic strategy of constructing the SMSI is to reduce metal nanoparticle (NP) precursors on the supports, such as metal oxides, metal sulfides, or even carbon-based materials, followed by heating at a high temperature. Due to the high surface energy on metal NPs and metal restructuring during the preparation or heterogeneous catalytic process, the SMSI has demonstrated efficient inhibition to the sintering, migration, or leaching of active metals and thus controlled the catalytic performance well, especially the stability. Although it is typically hard to observe the classical SMSI effect on carbon-supported catalysts due to the inert and non-reducible carbon support, several reports about the SMSI investigation have just been released recently [24, 27]. For instance, a strong chemical interaction between Pt NPs and sulfur-doped carbon supports was identified, which dramatically inhibits Pt overgrowth at moderate temperatures (300–700 °C) and then forms SMSI encapsulation structures on Pt NPs at a higher temperature (900–1000 °C) during annealing process [27]. It was further determined that the SMSI has a positive effect in promoting electrochemical ORR durability in H_2_–air fuel cells.

Since it has been widely accepted that the success of shape-controlled Pt-based NC synthesis is highly sensitive to many experimental factors (*e.g*., metal precursors, reaction conditions, and capping ligands), it remains a challenge to guide a harvest of various Pt-based NCs using a universal theory and methodology. Therefore, many research groups have developed their diverse synthetic protocols [2, 28, 29]. This review mainly covers the recent advances in general principles, shape-controlled mechanistic insight, established protocols, and corresponding electrochemical applications of Pt-based alloy/intermetallic NCs. Eventually, we outline the current challenges, perspectives, and developing trends in mechanisms and shape-control protocols based on the summary.

## General Principles for the Design of Pt-based Functional Electrocatalysts

NCs are typically synthesized using wet-chemistry methods referring to a bottom-up approach, which involves batches of solvents, precursors, capping ligands, and/or reducing agents. Additionally, the as-synthesized NPs are often stabilized or capped by surfactants in the solvents to confine their morphology, composition, and size distribution [1, 2]. Typical syntheses of Pt-based NCs prepared in a colloidal system using tungsten carbonyl (W(CO)_6_) were developed in the past decade, including Pt–Co, Pt–Ni, Pt–Fe, Pt–Cu bimetallic systems and Pt-based trimetallic systems [5, 17, 22, 30–33]. In such a case, W(CO)_6_ serves both functions of reducing and shape-control by in situ generating metallic tungsten and reductive carbon monoxide (CO) molecules through its thermal decomposition. It seems that CO can be preferentially adsorbed on Pt(100) and promote the evolution of Pt(100) [34]. Similarly, iron pentacarbonyl (Fe(CO)_5_) can also be utilized for synthesizing Pt–Fe NCbs, where the Fe metal atoms and CO are generated directly from the decomposition of Fe(CO)_5_ at a suitable temperature range [35, 36]. It was also reported that the sizes and morphologies of NCs could be modulated by the ratio of oleylamine (OAm) to oleic acid (OA), which are typically used as the solvents and/or capping ligands [37, 38]. For example, with decreasing the OAm/OA volume ratio from 10:1 to 4:1, a nano-concave feature can be evolved into a flat-like NP surface, indicating the overall reduction kinetics are pivotal in the shape control at a high OAm/OA ratio [32]. It is worth mentioning that in order to take into account a colloidal synthesis system, more variables (such as metallic valence in precursor, the ratio of different metal precursors, the capability of solvents, and reaction temperature) than the factors discussed above need to be considered from case to case [22, 39, 40]. Some traceable theoretical principles can guide the shape-controlled synthesis of colloidal metal NCs, which will be discussed from the thermodynamic and kinetic aspects.

### Synthetic Mechanism: Thermodynamic Control *vs*. Kinetic Control

**Fig. 1:**
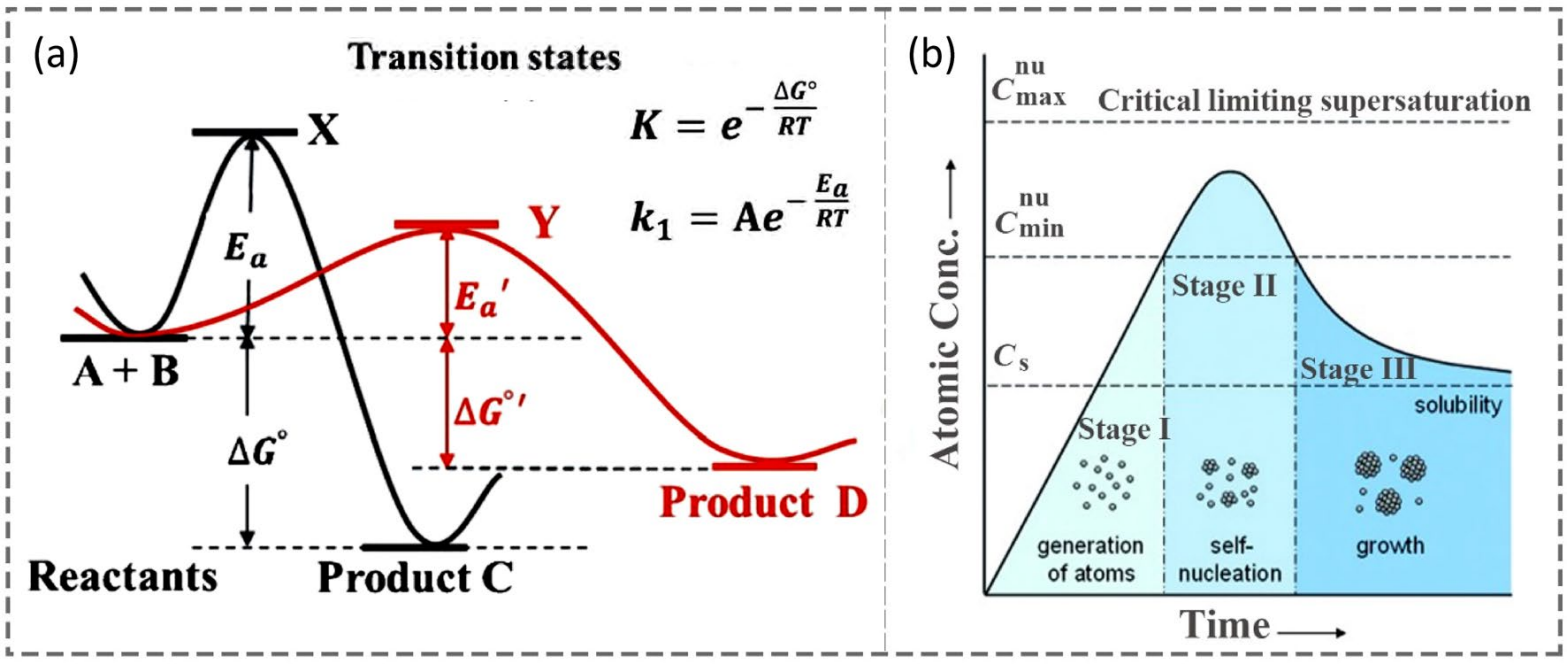
Energy and LaMer diagrams. **a** simple energy landscape, illustrating two reaction pathways controlled thermodynamically (in black) *vs*. kinetically (in red). Adapted with permission from Ref. [41] and modified. Copyright 2015 Wiley-VCH. **b** Idealized LaMer diagram, indicating the generation of atoms, nucleation, and subsequent growth as a function of time. Reproduced with permission from Ref. [4]. Copyright 2009 Wiley-VCH

To control a chemical reaction, it is necessary to understand its principles and mechanisms with thermodynamic and kinetic control. Typically, thermodynamics controls the driving force of a reaction originating from the difference between the initial and final states, while kinetics governs the energy barriers in the reaction pathway when it happens [29, 41]. In order to receive the desired product in a high yield, one needs to figure out whether the product is in the most stable state under thermodynamic control or it has the most favorable energy consideration within the reaction process (kinetic control). For example, Fig. 1a gives a simple schematic illustration in which reactants of A and B involve two parallel reaction pathways (A + B ↔ C, or A + B ↔ D) [41]. In such a case, product C is thermodynamically favorable through pathway one (in black), whereas D is the kinetic-control product via pathway two (in red). The reaction rate constant (*k*) is determined by the Arrhenius equation (Fig. 1a), which indicates that the higher temperature (*T*) is, the faster the reaction will be. Thus, the reaction temperature controls the conversion yields of products C and D. In other words, a low temperature favors the generation of product D with a fast reaction rate, whereas a high temperature promotes the formation of product C after establishing the equilibrium in the system. Although the thermodynamically controlled products are generally obtained with a global minimum of Gibbs free energy, the product during synthesis could be easily trapped in many states corresponding to local minima under kinetically controlled conditions. Importantly, temperature still plays the significant key role in determining thermodynamic or kinetic products. In this case, it is reasonable to conduct the synthesis at a relatively low or high temperature to fabricate either kinetically or thermodynamically controlled products. Considering the colloidal synthesis of NCs as one chemical reaction or a set of reactions, the abovementioned concept can be applied to the development of size, morphology, composition, and structure control using the lever of thermodynamics and kinetics. In this section, we will discuss the thermodynamic- and kinetic-controlled synthesis of NCs at the atomic level.

#### Thermodynamic Control

In general, the thermodynamic control in a bulk reaction can be simply determined by the total free Gibbs energy change ($\Delta {G}_{\mathrm{bulk}}$) driven by the starting state and final state [41]. Typically, $\Delta {G}_{\mathrm{bulk}}$ in bulk is negative when products are energetically more favorable than the reactants. Equation (1) indicates that the equilibrium depends on the overall enthalpy change ($\Delta H$), entropy change ($\Delta S$), and temperature ($T$). If both $\Delta H$ and $\Delta S$ are negative for the reaction, $\Delta {G}_{\mathrm{bulk}}$ will be dominated by $\Delta H$ at low temperatures and by $-T\Delta S$ at high temperatures in order to allow the reaction process.

1 $$\Delta {G}_{\mathrm{bulk}}=\Delta H - T \Delta S$$

In a nanoscale system, the surface energy $\int {\gamma }_{hkl}\mathrm{d}{A}_{hkl}$) must be considered accordingly, which is generally the key dominant factor in the NC synthesis. Equation (2) presents a typically modified Gibbs’ free energy:

2 $$\Delta {G}_{\mathrm{nano}}=\Delta {G}_{\mathrm{bulk}}+\int {\gamma }_{hkl}\mathrm{d}{A}_{hkl}$$

where the ${\gamma }_{hkl}$ is the specific surface free energy of a given crystallographic facet and $A_{hkl}$ is its surface area [4]. For liquid droplets or amorphous NPs, the ${\gamma }_{hkl}$ is independent of the direction as they are isotropic, but facets of anisotropic NCs usually have specific surface free energies because they have different surface ligands, coordination numbers (CNs), and charges. This makes it possible to control the size and morphology of NCs. For instance, without considering the capping ligand and other factors, an ideal facet is more stable in a given NC when its surface atoms have the largest CN. The ${\gamma }_{hkl}$ of the low-index facets on face-centered cubic (*fcc*) metal NCs generally follow the order of ${\gamma }_{(111)}< {\gamma }_{(100)}< {\gamma }_{(110)}$, implying that the (111)-facet formation is more energetic and favorable than the two other types of facets. For a body-centered cubic (*bcc*) structure of metallic NCs, interestingly, the order is altered as ${\gamma }_{(110)}< {\gamma }_{(100)}< {\gamma }_{(111)}$ [1, 4, 29]. In a colloidal synthesis, other factors, such as defect, hybrid, and surface-to-volume ratio (*S*/*V*), should also be considered synergistically. This makes thermodynamic control more complex [42, 43]. Nevertheless, this concept guilds the NC synthesis in general, allowing a possibility to predict and identify the dominant driving force theoretically and experimentally.

#### Kinetic Control

Unlike thermodynamics which solely relies on the starting state and ending state, kinetic control of a bulk chemical reaction is dramatically influenced by various factors within a set of elemental steps [44]. For example, there exists a kinetic barrier to generate nuclei in a homogeneous solution in the classic theory of the LaMer model [4]. Figure 1b illustrates the proposed stages in the synthesis of metallic NCs, including the supplied atoms from precursors (stage I), nucleation (stage II), and seeds growth (stage III) [45]. At the initial stage (Stage I), metal atoms are generated from chemical reactions (such as reduction or decomposition), and the concentration of atoms accumulates gradually up on the reaction time. During this stage, it allows the concentration exceeds the level of saturation (${C}_{s}$) without forming any stable nuclei. Once the concentration of atoms reaches the floor of supersaturating (designated as ${C}_{\mathrm{min}}^{\mathrm{nu}}$), stable seeds will be generated through homogeneous nucleation after overcoming the energetic barrier. Normally Stage II lasts for a very short period as these nuclei seeds consume large amounts of metal atoms, leading to a fast drop in the concentration of atoms below the level of ${C}_{\mathrm{min}}^{\mathrm{nu}}$. As a result, no additional nuclei will be created due to the lack of supersaturated atoms at this moment, and the residue metal atoms in the system would prefer to deposit onto the existing nuclei. These nucleated seeds will finally grow into NCs with controlled size and morphology in Stage III. Kinetic control is involved in each stage, that is, the reducing rate of metal atoms in Stage I, the energetic barrier of nuclei in Stage II, and the deposition rate versus the reducing rate in Stage III.

It has been wildly recognized that seed-mediated growth can be facilitated for shape control. In such a case, both the atom deposition rate (${V}_{\mathrm{deposition}}$) and surface diffusion rate (${V}_{\mathrm{diffusion}}$) are key kinetic parameters and can be manipulated by tuning the reaction conditions to harvest the desired kinetic products. The ${V}_{\mathrm{deposition}}$ can be expressed in Eq. (3):

3 $${V}_{\mathrm{deposition}}=k {[A]}^{x}{[B]}^{y}$$

where [*A*] stands for the concentration of a metal precursor A; [*B*] is the concentration of another reagent *B* (such as a reducing agent), *x* and *y* are the reaction orders of *A* and *B*, respectively; and *k* is the rate constant. The ${V}_{\mathrm{deposition}}$ can be controlled in several options, including adjusting [*A*], [*B*], altering the reaction temperature, and changing *x* and *y* by adopting different types of precursors or the other reagent. On the other hand, ${V}_{\mathrm{diffusion}}$ is another kinetic parameter that can be determined by the diffusion coefficient (*D*) that can be calculated as expressed in Eq. (4) [29]:

4 $$D={D}_{0} {\mathrm{e}}^{(-{E}_{\mathrm{diffusion}}/\mathrm{RT})}$$

where ${D}_{0}$ is the diffusion factor, ${E}_{\mathrm{diffusion}}$ is the energy barrier of the random atomic jumping, *R* is the ideal gas constant, and *T* is the reaction temperature. It is obvious that ${V}_{\mathrm{diffusion}}$ is mainly determined by the *T* and ${E}_{\mathrm{diffusion}}$, whereas ${E}_{\mathrm{diffusion}}$ involves the bonding energy (*BE*) between the surface atom to its neighbors and the surface functional groups (*i.e.*, capping ligand).

**Fig. 2:**
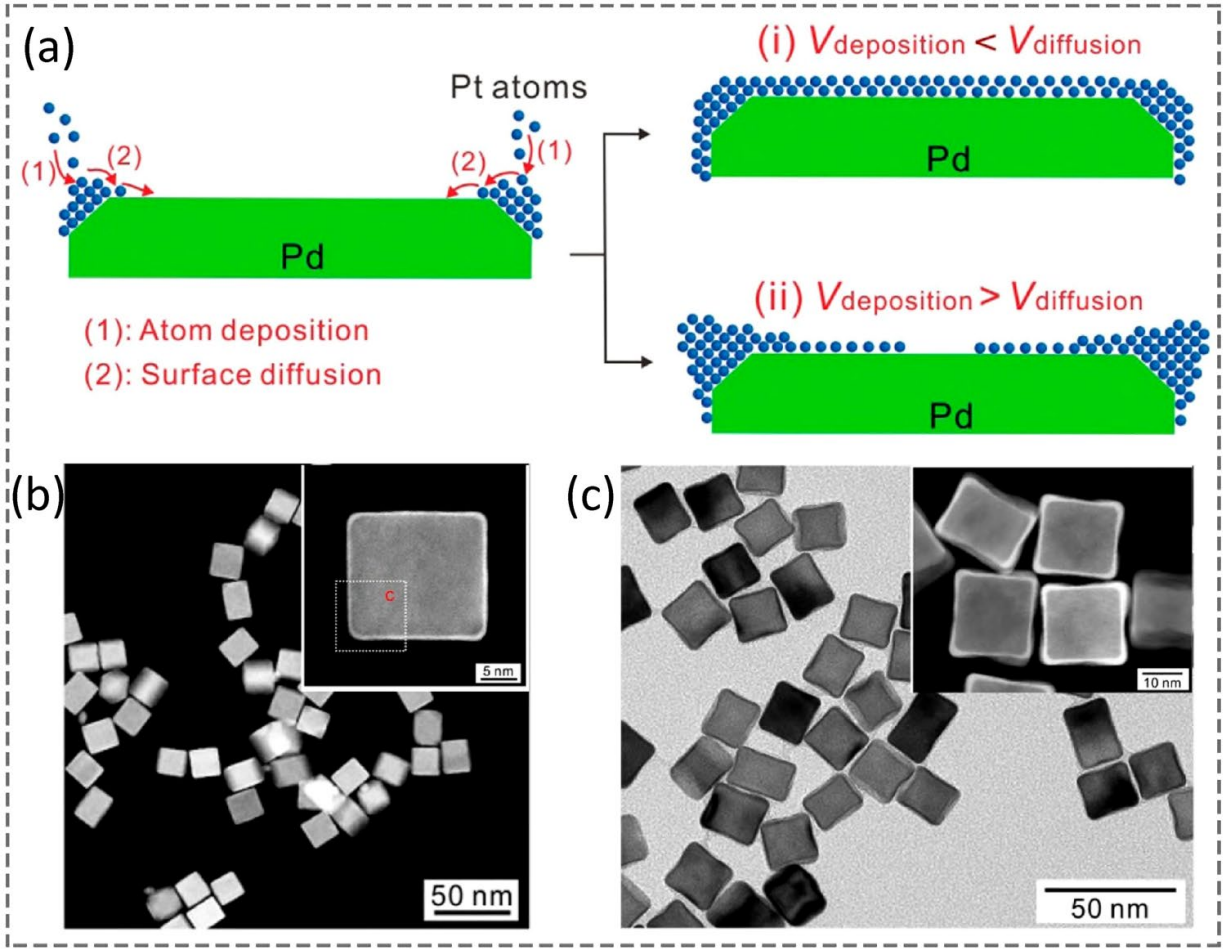
Mechanistic study of Pt-atom deposition on Pd nanocubes. **a** A schematic illustration, showing Pt-atom deposition on Pd cubic seeds through deposition and surface diffusion steps. **b** High-angle annular dark-field scanning transmission electron microscopy (HAADF-STEM) image of the Pd–Pt nanocube (the inset shows the zoom-in view of an individual Pd–Pt nanocube with a scale bar of 5 nm). **c** HAADF-STEM image of Pd–Pt concave nanocubes (the inset shows the zoom-in view of Pd–Pt concave nanocubes with a scale bar of 10 nm). Reproduced with permission from Ref. [46]. Copyright 2014 American Chemical Society

Taking the shape-controlled synthesis of palladium@platinum (Pd@Pt) core@shell NCs as an example [46], Fig. 2a illustrates the mechanism of forming Pd@Pt NCbs and concave NCbs with kinetic control. Initially, Pt atoms were deposited at the corners/edges on the Pd cores owing to the high local surface energy. The growth pathway strongly depends on the ratio of ${V}_{\mathrm{deposition}}/{V}_{\mathrm{diffusion}}$. If ${V}_{\mathrm{deposition}}<{V}_{\mathrm{diffusion}}$, most of the deposited atoms at the corners migrated to the facets adequately, and the epitaxial growth would prevail along < 100 > direction, producing Pd@Pt NCbs (Fig. 2b). In contrast, if ${V}_{\mathrm{deposition}}>{V}_{\mathrm{diffusion}}$, most of the Pt atoms remained at the corners/edges, and Pd@Pt concave NCbs were developed alternatively (Fig. 2c). Similarly, this strategy can be extended to other Wulff shapes of NCs, such as octahedra, decahedra, and icosahedra [3].

### Morphology Control: Growth Mechanism and Assessment

In order to design functional NCs with morphology control, it is critical to understand how their facets develop in the synthesis. Tracing back to 1873, Gibbs proposed that the equilibrium shape of a droplet of matter is determined by surface energy minimization. Later on, when Wulff construction was used to predict the equilibrium shape of NCs [47], the surface energy was considered to be proportional to a normal vector drawn from the crystal center to an external surface. This model can be rationally extended from liquid droplets to solid NCs. Generally, the growth rate in a direction perpendicular to a high-surface-energy facet is faster than that to the low-surface-energy facet. Thus, the high-surface-energy facets will eventually disappear, leaving the low-surface-energy facets terminated on the NCs. Note that, in practice, the surface energy of a facet cannot be simply determined using the “ideal” order given in Sect. 2.1.1 which is usually determined under a vacuum condition. In a colloidal synthesis, solvents and surfactants are commonly used to modify the total surface energy by preferential adsorption on specific facets, regulating the relative growth rates in directions perpendicular to different facets to realize the shape control of NCs. Although several thermodynamic- and kinetic-based mechanisms have been proposed, the facet development at the atomic scale still remains mysterious primarily due to the lack of in situ/*Operando* observation.

**Fig. 3:**
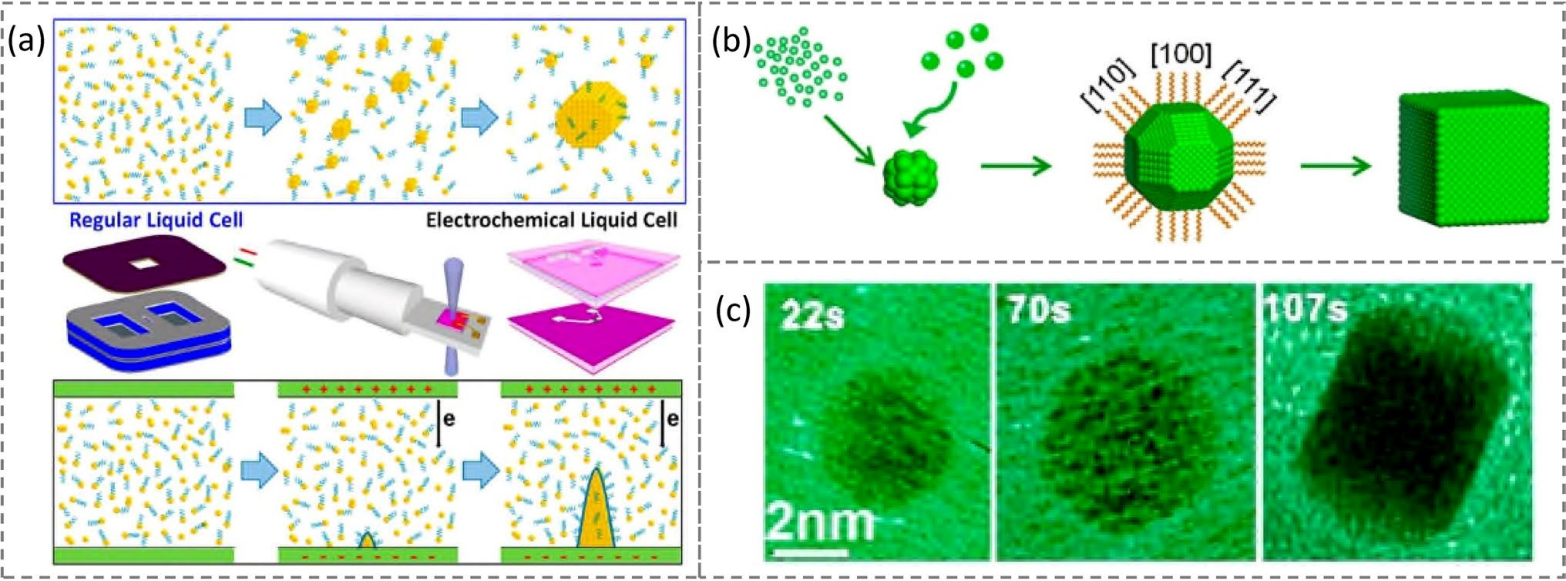
The facet development of a Pt nanocube. **a** Schematic overview of TEM visualization of colloidal nanocrystal growth and electrochemical liquid reactions. **b** Schematic illustration of the growth of a nanocube in a liquid solution. **c** Real-time imaging of the growth of Pt nanocube using the liquid cell. Reproduced with permission from Ref. [54] and modified. Copyright 2017 American Chemical Society

Thanks to the fast development of microfabricated liquid cells equipped with transmission electron microscopy (TEM) with a high spatial and temporal resolution (Fig. 3a), making it possible to directly monitor the nucleation and subsequent evolution of the facets of NCs [48–52] (*i.e.*, Stages II and III in Fig. 1b). For example, the Livisatos group has observed two different growth pathways using *in *situ liquid cell TEM [51, 52]. They determined that Pt NCs can grow either by monomer attachment from solution or by seed coalescence, suggesting that colloidal NCs take different growth pathways due to the differentials of surface energies based on their size- and morphology-dependent internal energies. Using a liquid cell TEM technique, the Zheng group, later on, imaged a real-time growth of Pt_3_Fe nanorods from solution-based NC building blocks, which experienced shape-directed NP attachment, straightening, orientation, and shape corrections [53]. They subsequently reported their observation on one Pt NCb growth as well [49] by monitoring the real-time shape-development. By focusing on an individual Pt NP growth process (Fig. 3b), it was found that the initial growth rates of low-index facets are similar during the nucleation (Stages I and II) until the {100} facets stop the growth (beginning of Stage III) [54]. The successive growth (Stage III) of the rest facets (especially the {111} facets) leads to the final evolution of {100}-terminated Pt NCb (Fig. 3c).

The in situ liquid cell TEM can also be extensively applied to monitoring the preferential etching and, thus, shaping of NCs using a top-down strategy [55–57]. For example, the elective adsorption of surface ligands associated with their inhibiting behavior was observed through the etching of Pd NCs [58]. It revealed that the etching rate could be exclusively controlled by adjusting the concentrations of ligands. At a low ligand concentration (0.1 mM iron acetylacetonate (Fe(acac)_3_)), a rapid etching primarily occurs at (100) facets, resulting in concave NCs, whereas at a high ligand concentration, the etching rate is reduced due to the sufficient ligand coverage on (100)-facets, eventually evolving round NCs. Both the direct investigation and Ab initio calculation suggested that the differences in the adsorption energy of inhibitor molecules on Pd facets were responsible for the etching control.

## General Synthetic Strategies for Pt-Based Nanocrystals

Approaches to fabricating metal NCs in a colloidal system can be fundamentally classified into two categories: (1) top-down and (2) bottom-up. The top-down strategy is to reduce the bulk materials to nanoscale dimensions using physical or chemical methods. In contrast, the bottom-up design involves the construction of NCs from nucleation, deposition, and assembly of atoms, molecules, or clusters [59–61]. By adopting physical techniques such as optical lithography, electron beam lithography, and ball milling process [62–64], the top-down physical strategy is beneficial to fabricate nano-patterns and large-quantity NPs. With kinetic energy provided by the ball milling, for example, bulk materials can be ground into NPs in high yield [60]. However, the as-prepared NPs might be inferior due to the significant damage to their crystallographic surfaces. Another universal approach under the top-down category is chemical engraving (such as selective de-alloying and template etching) and emulsification, to produce the nanostructures [65]. For instance, de-alloying is generally used to remove one metal component from metal alloy NCs, producing porous nanostructures, nanocages, or nanoframes (NFs) [39, 66, 67]. The Strasser group has systematically investigated the de-alloying process (acidic de-alloying process or electrochemical leaching process) in Pt–Cu, Pt–Co, and Pt–Ni systems and reported that the morphologies and nanostructures of the de-alloyed products highly rely on their sizes [68–70]. For example, individual core–shell Pt–Co@Pt and Pt–Cu@Pt NPs with a Pt-rich shell were exclusively observed after a de-alloying process if the size (diameter) is less than 10–15 nm. For those with a size between 10–15 and 30 nm, irregularly shaped structures could be generated after this process. For Pt–Co and Pt–Cu NPs with a diameter (of multiple cores) over 30 nm, nanoporous structures on the surface of Pt–Co NCs were determined [69].

In contrast to the top-down strategy, the bottom-up design has been much more commonly used to precisely control NCs in size, composition, and morphology [4, 60, 71, 72]. Various methods, such as colloidal synthesis, microwave-assisted synthesis, and thermal pyrolysis, have been established [1]. In addition, by combining the “bottom-up” and “top-down” subsequently, nanohollows, frameworks, and nanocages of metals or metal alloys have been prepared with fascinating properties [73]. For example, a protocol of de-alloying PtNi_4_ tetrahexahedral (THH) NCs into high-indexed Pt_3_Ni NFs through a Mond process consisting of two stages has been developed [67], in which PtNi_4_ THH NCs were first synthesized from a hot colloidal solution, and the Ni atoms in the NCs were then selectively etched along the < 100 > orientation using carbon monoxide (CO), generating gaseous Ni(CO)_4_. The resultant Pt_3_Ni alloy THH NFs contain segregated Pt thin layers with compressive strain and exhibit much improved electrocatalytic performance toward ORR. Similarly, there have been many other achievements using combined synthetic approaches. In this section, recent progress in the morphology-controlled synthesis of Pt-based alloy and intermetallic compounds is centralized.

### Morphology Control of Pt NCs

**Fig. 4:**
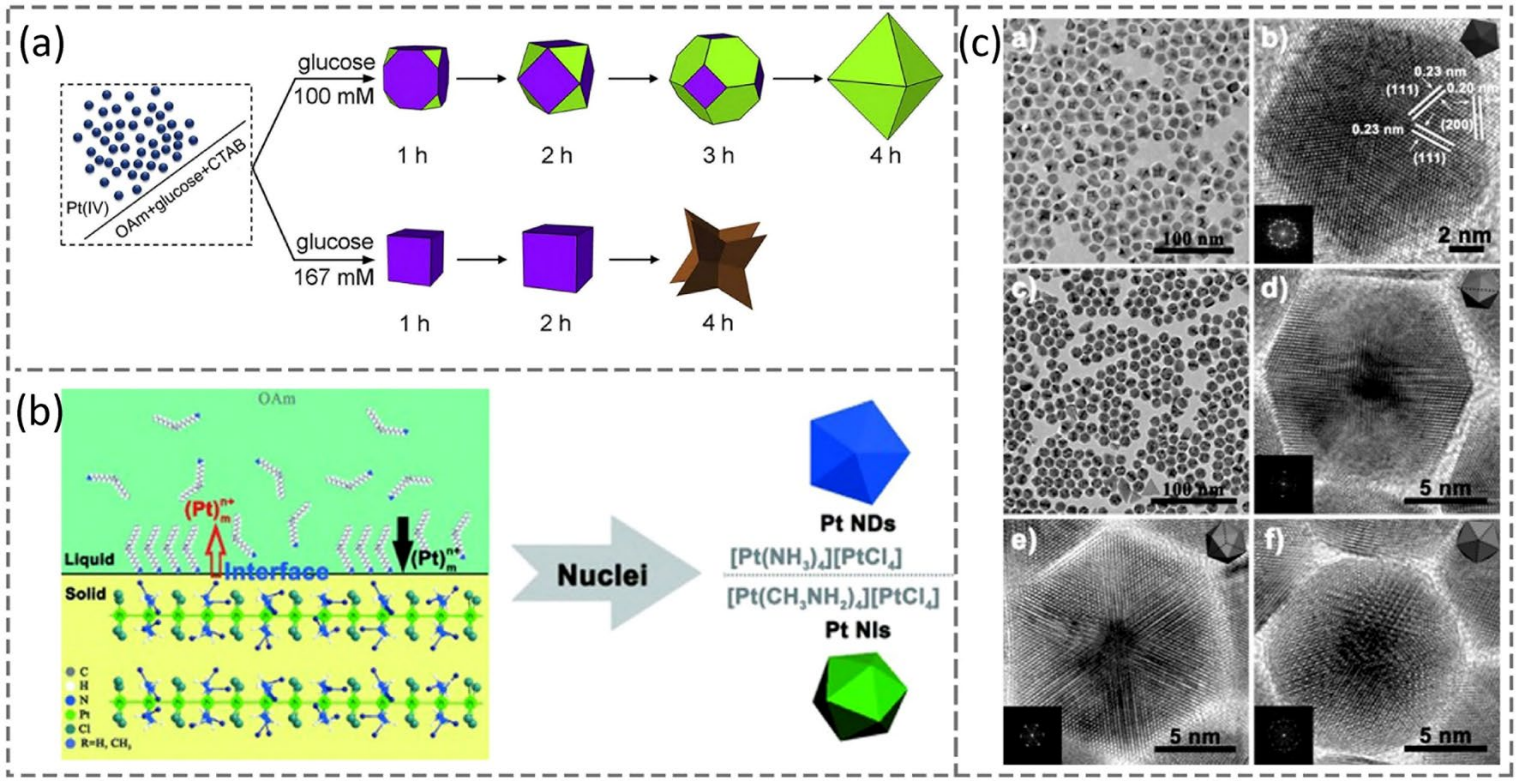
**a** Schematic illustration of two different growing pathways of Pt nanocrystals with distinctive shapes as a function of reaction time. Reproduced with permission from Ref. [77] and modified. Copyright 2018, Elsevier. **b** Growth mechanism of Pt nanodecahedra and nanoicosahedra. **c** HRTEM images of Pt nanodecahedra and nanoicosahedra with incident direction on two/three/fivefold axis. Reproduced with permission from Ref. [78]. Copyright 2012 Wiley-VCH

The shape-controlled syntheses of metallic NCs are usually implemented through the following routes: (1) direct one-pot synthesis with homogeneous nucleation, (2) seed-mediated epitaxial growth, and (3) galvanic replacement [1, 74]. The mechanism of the first route has been discussed in Sect. 2.1.2, and this method could generate Pt NCs in various morphologies (*i.e.*, NCb, octahedron, decahedron, icosahedron, and tetrahexahedron), exposing with low- or high-index crystallographic facets, as summarized in Table 1. Typically, Pt NCs are terminated with low-index facets such as {100}, {111}, and {110} due to their relatively low specific surface free energies. It should also point out that the homogenous reaction media is usually applied to these syntheses but not the only cases. For example, the Fang group reported a synthesis of Pt NCbs using tungsten hexacarbonyl (W(CO)_6_) as the reducing agent [31]. It was identified that W(CO)_6_ is crucial to control the Pt-atom generation and nucleation processes (Stages I and II). It is believed that the tungsten (W) decomposed from W(CO)_6_ can facilitate the reduction of Pt precursors with its low reduction potential, regulate the feedstock of Pt atoms, and lead to a fast Pt nucleation, whereas the CO decomposed from W(CO)_6_ can stabilize the {100} facets of Pt seeds through its preferential adsorption on Pt {100} facets, preserving these facets in Stage II before the capping ligands such as OAm and OA maintain these facets during the crystal growth (Stage III). By replacing W(CO)_6_ with other reducing agents such as Mn_2_(CO)_10_ and H_2_, Pt NCbs can also be yielded at an optimized reaction temperature. It was even reported that CO can solely facilitate Pt NCbs without the assistance of other capping ligands [34]. However, this single CO protocol cannot produce binary Pt-alloy NCbs (vide infra) [75]. Since the specific surface free energy of Pt(111) (${\gamma }_{(111)})$ is lower than that of Pt(100) ( ${\gamma }_{(100)})$ intrinsically, octahedral Pt NCs should be more stable thermodynamically than Pt NCbs if ${\gamma }_{(111)}$ and ${\gamma }_{(100)}$ would not be significantly changed in a colloidal system. Thus, by lowering the concentration of the reducing agent and adjusting reaction time, Yang and Xia groups have successfully demonstrated their syntheses of truncated cubes, cuboctahedra, truncated octahedra, octahedra, cubes, and concave Pt NCs, respectively [76, 77]. As depicted in Fig. 4a, Pt NCs in various shapes could be readily generated at different reaction-time scales [77]. When the synthesis was carried out with glucose at a low concentration of reducing agent (glucose, 0.1 M), a series NCs enclosed with a mixture of (100)- and (111)-facets can be produced after 1, 2, and 3 h, whereas sufficient reaction time (*e.g.*, 4 h) could lead to perfect octahedral Pt NCs. Alternatively, by increasing glucose concentration to 0.167 M, Pt NCbs, larger NCbs, and concave NCbs could be obtained after 1, 2, and 4 h, respectively. This indicates that the reduction kinetics, as determined by the glucose concentration, is largely responsible for the diversified Pt NCs in shape and size.

**Table 1:** Summary of some synthesized Pt NCs in various morphologies

Pt	Morphology	Preparation condition	References
Low-index	Cube	Pt(acac)_2_ + W(CO)_6_ at 240 °C	[31]
		Pt(acac)_2_ + Mn_2_(CO)_10_ or CO at 240 °C	[81]
	Cube	Pt(acac)_2_ + OAm/OA + Fe(CO)_5 (trace amount)_ at 200 °C	[82]
	Cube, rod, pod	Pt(acac)_2_ + H_2_ from 40 to 100 °C	[83]
	Octahedron	Na_2_PtCl_6_ + PVP at 990 °C	[77, 84]
		Pt(acac)_2_ + Mn_2_(CO)_10_ at 210–230 °C	[81]
High-index	Concave	Na_2_PtCl_6_ + glucose + CTAB at 160 °C	[77]
	Decahedron	[Pt(NH_3_)_4_][PtCl_4_] + OAm at RT	[78]
	Icosahedron	[Pt(CH_3_NH_2_)_4_][PtCl_4_] + OAm at 150 °C	[78]
		Pt(acac)_2_ + Mn_2_(CO)_10_ at 190–210 °C by size-selective precipitation	[81]
		Pt(acac)_2_ + Y(acac)_2_ + CO at 210 °C by the hot injection	[85]
	Tetrahexahedron	Pt nanospheres + 30 mM ascorbic acid + 0.1 M H_2_SO_4_ under the specific square-wave potential range	[79]
		Sb, Bi, Pb, and Te + Pt precursors at 900 °C under Ar/H_2_	[80]

*OAm* oleylamine, *OA* oelic acid, *acac* acetylacetonate, *CTAB* hexadecyltrimethylammonium bromide *PVP* poly(vinyl pyrrolidone)

As tabulated in Table 1, Pt NCs enclosed with high-index facets, such as concave cubes, decahedra, icosahedra, and tetrahexahedra, were also prepared successfully. The Yan group reported their syntheses of Pt nanodecahedra (NDs) and nanoicosahedra (NIs) through a reduction of Pt complexes [78], [Pt(NH_3_)_4_][PtCl_4_] and [Pt(CH_3_NH_2_)_4_][PtCl_4_]. The success is attributed to the unique structure of the linear Pt(II) complex precursors that possess strong metal–metal interactions and their long slow reduction rates, which were identified as the prerequisites to the formation of Pt multiply twinned particles, as illustrated in Fig. 4b. TEM images of these NDs and NIs are displayed in Fig. 4c. The Sun group has demonstrated their preparation of Pt-THH NCs (*ca.* 200 nm in size) in a high yield through an electrochemical treatment on Pt nanospheres supported on glassy carbon using square-wave potentials [79]. The single crystal THH NC is enclosed by 24 high-index and high-energy facets. The Mirkin group also reported their preparation of Pt-THH NCs (10–500 nm) using a ligand-free solid-state reaction method [80]. This alloying-dealloying (shape–regulating) process is distinct from the classical additive-growth process in a colloidal system. Pt-based THH NCs were first generated on silicon wafers or other catalytic supports by heating the metal precursor in a tube furnace with atmosphere from a trace amount of foreign elements (such as antimony (Sb), bismuth (Bi), lead (Pb), or tellurium (Te)) to stabilize high-index facets ((210) planes). Then, the in situ de-alloying process of the trace element (e.g*e.g*., Sb) occurred kinetically from the initial Pt–Sb alloy NCs, eventually producing Pt-THH NCs.

### Morphology Control of Pt-Alloy NCs

**Fig. 5:**
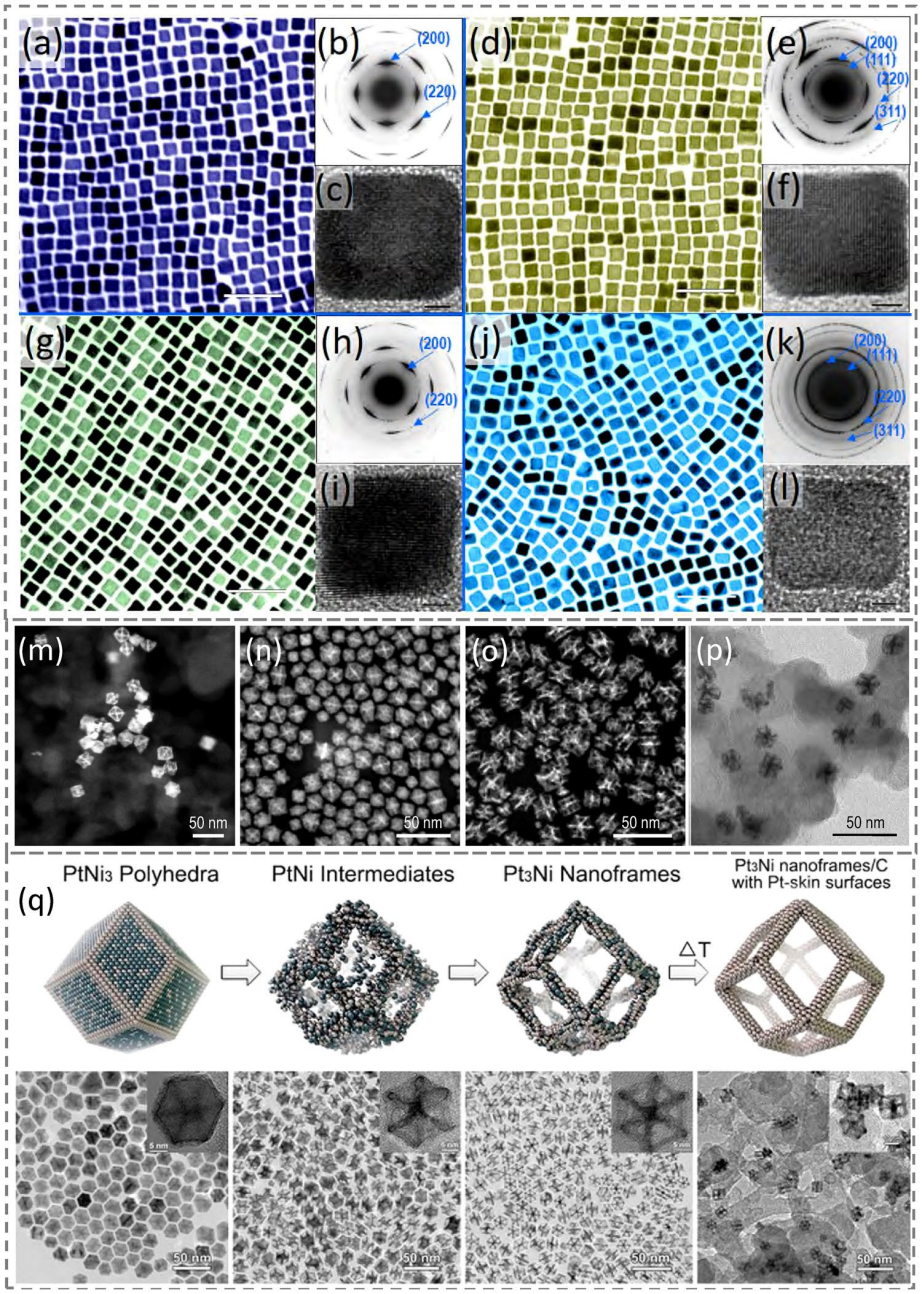
TEM images, diffraction patterns, and schematic illustrations of Pt-based nanocrystals. **a–c** Pt nanocubes. **d–f** Pt_3_Co nanocubes. **g–i** Pt_3_Fe nanocubes. **j–l** Pt_3_Ni nanocubes. **a**, **d**, **g** and **j** show TEM images (data bars represent 50 nm); **b**, **e**, **h** and **k** show the selected area electron diffraction (negative) patterns of the nanocubes; **c**, **f**, **i** and **l** show high-resolution TEM images of the NCbs (scale bars represent 2 nm). Adapted with permission from Ref. [31] and modified. Copyright 2009 American Chemical Society. **m–o** HAADF-STEM images of nanoframes. **m** Pt_3_Ni tetrahexahedral nanoframes supported on carbon and evolved through CO thermal annealing. Reproduced with permission from Ref. [67] and modified. Copyright 2017 American Chemical Society. **n** Pt–Ni tetrahexahedral nanoframes generated by etching Pt–Ni tetrahexahedral nanocrystals using acetic acid at 100 °C overnight under stirring. **o** Pt–Ni rhombic dodecahedral nanoframes produced by etching Pt–Ni rhombic dodecahedral nanocrystals using acetic acid at 100 °C overnight under stirring. Reproduced with permission from Ref. [87] and modified. Copyright 2016 American Chemical Society. **p** TEM image of Pt–Co rhombic dodecahedral nanoframes yielded by etching Pt–Co RD nanocrystals using 2 M HNO_3_ under vigorous stirring at 60 °C for 1 h in air. Reproduced with permission from Ref. [88] and modified. Copyright 2020 American Chemical Society. **q** Schematic illustrations of the interior erosion stages and corresponding TEM images in each representative stage of the Pt_3_Ni rhombic dodecahedral nanoframe development. Reproduced with permission from Ref. [89]. Copyright 2020 American Chemical Society

It has been widely recognized that some physicochemical properties of Pt-alloy NCs are superior to those of pure Pt NCs [1, 3]. Pt-alloy NCs are usually prepared through the first synthesis route discussed in Sect. 3.1 using either a combination of reduction-thermal decomposition or co-reductions of precursors. For example, monodisperse Pt-Fe NCs were first synthesized as early as 2000 by the reduction of platinum acetylacetonate coupled with a decomposition of iron pentacarbonyl in the presence of OAm and OA [36]. After altering the reaction solvent and optimizing other conditions of this synthesis strategy, the successful preparation of Pt-Fe NCbs was shortly reported by the Sun group [35]. Alternatively, Pt_3_Co, Pt_3_Fe, and Pt_3_Ni NCbs can also be co-reduced in a hot organic solution in the presence of capping ligands and W(CO)_6_, using the same approach [31] discussed in Sect. 3.1. As shown in Fig. 5a–l, Pt, Pt_3_Co, Pt_3_Fe, and Pt_3_Ni NCbs prepared through this co-reduction strategy present perfect cubic morphology. Their corresponding selected area electron diffraction (SAED) patterns and HRTEM images confirm the structures and high crystallinity. Significantly, Pt_3_Cu_2_ [40, 86] and Pt_3_Ni [17] nano-octahedra have also been fabricated by adjusting the reaction conditions using this synthesis strategy.

Besides Pt-based NCs with low-index facets, high-index Pt-based NCs can also be synthesized either in a one-pot colloidal solution [32, 67] or by a multi-step protocol [39, 67, 87]. For example, monodisperse PtNi_4_ THH NCs with well-defined (730) facets were synthesized by co-reducing PtCl_4_ and NiCl_2_·6H_2_O in the presence of OAm in 1-octadecene [67]. The temperature remained at 180 °C for ~ 10 min first to generate Pt-based seeds, followed by fast heating to 290 °C within 5 min and quenching immediately once reaching the desired temperature. Next, CO was used to de-alloy Ni components from PtNi_4_ THH NCs at 170 °C, resulting in Pt_3_Ni THH NFs (Fig. 5m). Alternatively, Pt–Ni THH NCs can be synthesized by co-reducing Pt(II) and Ni(II) acetylacetonate in the presence of dodecyltrimethylammonium chloride, OAm, and OA at 180 °C for 3 h [87]. By adjusting the fractions of OAm and OA, Pt–Ni rhombic dodecahedra (RD) can be produced in a similar system. Subsequently, these Pt–Ni THH and RD NCs can be etched with acetic acid at 100 °C for 10 h and converted to THH and RD NFs (Fig. 5n–o). Using a similar strategy, Pt–Co RD NFs (Fig. 5p) were synthesized by etching Pt–Co RD NCs using 2 M HNO_3_ at 60 °C under vigorous stirring for 1 h in air. The resultant Pt–Co RD NFs showed higher activity toward ORR than their Pt–Ni counterparts [88]. Earlier, it was reported that OAm-capped PtNi_3_ RD NCs could transform into hollow Pt_3_Ni NFs in solution by either keeping them in nonpolar solvents such as hexane and chloroform under ambient conditions for 2 weeks or heating them in a similar system at 120 °C for 12 h [39]. A schematic illustration of the interior erosion stages and corresponding TEM images in each representative stage are shown in Fig. 5q. The resultant hollow 3D RD NFs possess Pt-rich composition on the surface of each edge and the high surface area-to-volume ratio [90], exhibiting improved performance toward ORR. It is expected that the aforementioned fabrication methods of NFs could be readily applied to other Pt-alloy systems, such as Pt–Cu, Pt–Co, Pt/Rh–Ni, and Pt/Pd–Ni. With different experimental conditions, Table 2 outlines some typical syntheses of Pt-alloy nano-polyhedra and NFs.

**Table 2:** Summary of some synthesized Pt-alloy NCs in various morphologies

Metal/alloy	Morphology	Composition	Preparation condition	Refs
Pt–Co	Cube	Pt_3_Co	Pt(acac)_2_ + Co(acac)_2_·4H_2_O + W(CO)_6_ at 240 °C	[31]
	Concave cube	Pt_3_Co	Pt(acac)_2_ + Co(Ac)_2_·4H_2_O + W(CO)_6_ with a high ratio of OAm/OA at 240 °C	[30]
	Excavated Octahedron	PtCo	Pt(acac)_2_ + Co(Ac)_2_·4H_2_O + CTAB	[91]
	Rhombic Dodecahedra (RD)	Pt_21_Co_79_	H_2_PtCl_6_·6H_2_O + Co(Ac)_2_·4H_2_O + OAm + OA at 240 °C	[88]
	RD NFs	Pt_83_Co_17_	Pt–Co RD + 2 M HNO_3_ at 60 °C	[88]
Pt–Fe	Cube	Pt_3_Fe	Pt(acac)_2_ + FeCl_2_ ·4H_2_O + W(CO)_6_ at 240 °C	[31]
	Cube	Pt-Fe	Pt(acac)_2_ + Fe(CO)_5_ + OAm/OA at 205 °C	[35]
	Concave cube	Pt_3_Fe	Pt(acac)_2_ + Fe(acac)_3_ + W(CO)_6_ at 240 °C	[32]
Pt–Ni	Cube	Pt_3_Ni	Pt(acac)_2_ + NiCl_2_·6H_2_O + W(CO)_6_ at 240 °C	[31]
	Octahedron	Pt_3_Ni	Pt(acac)_2_ + Ni(acac)_2_ + W(CO)_6_ at 240 °C	[17]
		Pt_1.5_Ni, PtNi, PtNi_1.5_	Pt(acac)_2_ + Ni(acac)_2_ + DMF at 120 °C	[92]
	Concave octahedron	Pt_3_Ni	1. Pt(acac)_2_ + Ni(acac)_2_ + PVP Ibenzoic acid at 150 °C 2. Pt–Ni alloy NCs + dimethylglyoxime + acetic acid	[73]
	Tetrahexahedron	PtNi_4_	PtCl_4_ + NiCl_2_·6H_2_O at 290 °C	[67]
	THH NF	Pt_3_Ni	PtNi_4_ THH + CO at 170 °C	[67]
	RD	PtNi_3_	H_2_PtCl_6_·6H_2_O + Ni(NO_3_)_2_·6H_2_O + OAm at 270 °C	[39]
	RD NFs	Pt_3_Ni	PtNi_3_ RD + O_2_ etching	[39]
Pt–Cu	Cube	Pt_3_Cu	Pt(acac)_2_ + Cu(acac)_2_ + 1,2-tetradecanediol at 230 °C	[37]
	Octahedron	Pt_3_Cu_2_	Pt(acac)_2_ + CuCl + CuCl_2_ + W(CO)_6_ at 240 °C	[40]

*OAm* oleylamine, *OA* oleic acid, *acac* acetylacetonate *Ac* acetate, *PVP* poly(vinyl pyrrolidone), *DMF* dimethyl formamide

### Morphology Control of Pt-Based Core@Shell NCs and Pt-Based Nanocages

In opposition to the first route (directly one-pot synthesis) on a shape-controlled synthesis of Pt or Pt-alloy NCs, the second and third routes are commonly applied to the development of core@shell NCs. As discussed in Sect. 2.1.2, the products from a seed-mediated growth are typically governed by competition of thermodynamics and kinetics (*V*_deposition_ *vs*. *V*_diffusion_). The growth modes for thin film deposition and NCs can generally be broken into three types: (1) layer-by-layer deposition (Frank-van der Merwe Mode), (2) island growth (Volmer-Weber mode), and (3) the intermediate mode (Stranski–Krastanov mode). For instance, highly faceted cubic Pt seeds can be used to direct the shape-controlled formation of a secondary metal such as Pd or Au through the epitaxial overgrowth [93]. This concept led to the successful preparation of core@shell structured Pt@Pd NCs in different morphologies (NCbs, cuboctahedra, and octahedra) and Au nanorods on cubic Pt seeds. Subsequently, the preparation of monodisperse Au@Pd and Au@Ag core–shell NCbs through epitaxial growth on octahedral Au seeds was also reported [94]. Furthermore, it was claimed that the thickness of the shell layer could be controlled thermodynamically and kinetically [46, 95–98] on Pd@Pt core–shell NCs in various morphologies such as concave NCbs, nano-octahedra, truncated nano-octahedra, concave NDs, and NIs as summarized in Table 3. In addition, due to the lower standard reduction potential of Pd^2+^/Pd (0.951 V) than that of Pt^2+^/Pt (1.180 V), the Pd core can be selectively oxidized and etched out from the NCs by FeCl_3_/HCl or HNO_3_ in this approach, generating Pt-based nanocages in different morphologies (Table 3).

**Table 3:** Summary of some Pt-based core@shell NCs and Pt-based nanocages in various morphologies

	Morphology	Preparation condition	References
	*Core@shell NCs*		
Pd@Pt core@shell	Cube	Pd cubic seeds + AA + PVP + KBr + Na_2_PdCl_4_ at 80 °C	[46]
	Concave cube	Pd cubic seeds + PVP + KBr + H_2_PtCl_6_ at 90 °C	[99]
	Octahedron	Pd octahedral seeds + PVP + CA + KBr + K_2_PtCl_4_ at 95 °C	[98]
	Truncated Octahedron	Pd octahedral seeds + PVP + AA + KBr + EG + Na_2_PtCl_6_ at 200 °C	[98]
	Concave Decahedron	Pd decahedral seeds + PVP + AA + KBr + Na_2_PtCl_6_ at 200 °C	[100]
	Icosahedron	Pd icosahedral seeds + PVP + AA + KBr + Na_2_PtCl_6_ at 200 °C	[101]
	*Nanocage*		
Pt–Pd	Cubic cage	Pd@Pt core@shell NCbs + FeCl_3_/HCl etching	[97]
	Octahedral cage	Pd@Pt core@shell octahedra + FeCl_3_/HCl etching	[97]
	Icosahedral cage	Pd@Pt core@shell icosahedra + HNO_3_ etching	[102] [103]
	Concave Decahedron	Pd@Pt concave decahedra + FeCl_3_/HCl etching	[100]
Pt–Cu	Nanocage	H_2_PtCl_6_ + Cu(acac)_2_ + CTAB at 170 °C	[104]
Pt–Ni	Bunched Nanocage	Pt(acac)_2_ + Ni(acac)_2_ + CTAB at 180 °C + HNO_3_	[105]
PtCuPd@Ru	Yolk-cage	Pt-Cu core@shell nanoplates → growth with Ru → electrochemically etching of PdCu	[106]

*AA* ascorbic acid, *PVP* poly(vinyl pyrrolidone), *acac* acetylacetonate, *CTAB* hexadecyltrimethylammonium bromide

**Fig. 6:**
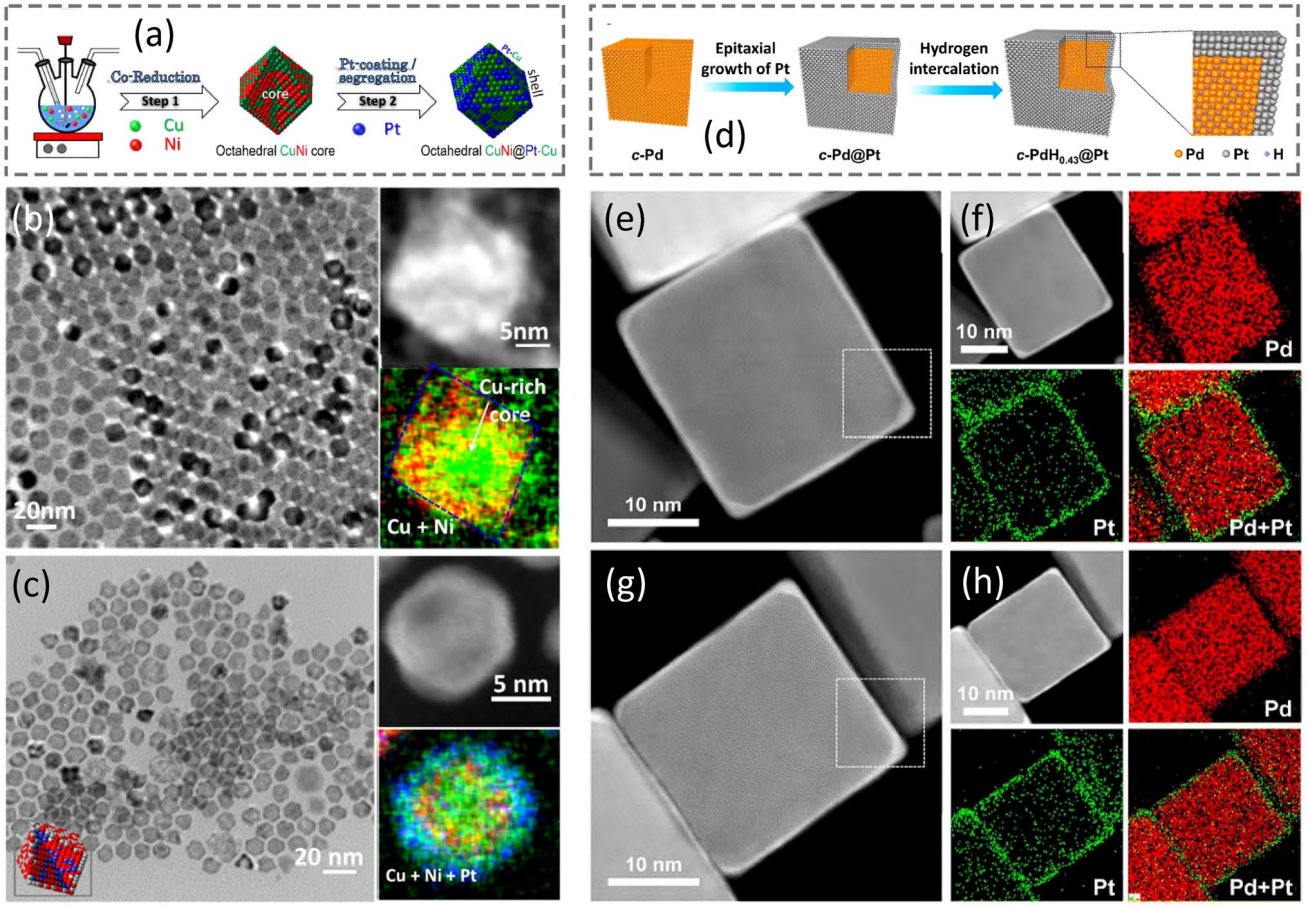
Synthesis of Pt-based core@shell nanocrystals. **a** Schematic illustration of CuNi@Pt–Cu core@shell nano-octahedra through a seed-mediated process. **b** TEM image, HADDF-STEM image, and corresponding EDX elemental map of CuNi nano-octahedron core. **c** TEM image, HAADF-STEM image, and corresponding EDX elemental map of CuNi@Pt–Cu core@shell nanostructures. **a**–**c** Adapted with permission from Ref. [3] and modified. Copyright 2021 Wiley-VCH. **d** Schematic illustration of Pd@Pt and PdH_0.43_@Pt core@shell NPs. **e, f** HAADF-STEM image, and corresponding EDX elemental mapping images of Pd@Pt nanoparticles. **g, h** HAADF-STEM image, and corresponding EDX elemental mapping images of PdH_0.43_@Pt core@shell nanoparticles. Adapted with permission from Ref. [109] and modified. Copyright 2021 American Chemical Society

It is challenging but interesting to choose a non-precious metal as the core seeds. A practical strategy is to develop the core@shell nanostructures using 3*d* transition metals such as Cu, Ni, or Fe as the cores [107]. One of the successful examples is the monodisperse core@shell Ni@FePt NPs via a seed-mediated growth with controlled shell thickness (~ 1 nm) [107]. The success in harvesting this kind of core@shell structured NPs highly relies on the interface of the Ni seeds. Oxidation on the surfaces of the Ni seeds would prevent uniform growth of Pt or Pt-alloy shell around them. Consequently, protection from oxidation on Ni seeds could favor the formation of core@shell nanostructures. Usually, the morphology of the resultant core@shell products was polyhedra rather than a single type of shape desired. Recently, a synthetic protocol of CuNi octahedral core-based CuNi@Pt–Cu core@shell NPs was developed [3, 108], consisting of a seed-mediated method coupled with selective galvanic replacement in a colloidal system (Fig. 6a). While the ratio of *V*_deposition_/*V*_diffusion_ was precisely tuned by optimizing the parameters such as the capping ligand, growth temperature, and the ramp rate of heating, uniform Pt-based shells could eventually grow on the CuNi octahedral cores (Fig. 6b) with a thickness of < 1 nm (Fig. 6c). The structure, morphology, and local composition of the resultant CuNi@Pt–Cu NPs have been confirmed by the TEM and HAADF images as well as the EDX maps (Fig. 6c). Considering the CuNi cores are subject to surface oxidation easily, the presence of the consecutive oxide shell would suppress the growth of the Pt-alloy shell on the core template. It was identified that more optimal reaction conditions are required in the 3*d*-transition-metal-core-based synthesis. For this reason, a fast ramp rate of heating is necessary to protect the intermediates from possible oxidation. However, a hasty heating rate (e.g*.*, > 6 °C min^−1^) would result in branched structures in the products since *V*_deposition_ >  > *V*_diffusion_. On the other hand, the Pt-precursor plays another role. For example, a replacement of PtCl_4_ with Pt(acac)_2_ would generate “core-satellite”-like nanostructures. Recently, the Zhang group also reported a hydrogen intercalation method to tune the lattice of Pt on specific Pt facets while Pd/PdH_0.43_ NCs were used as the cores (Fig. 6d), and thus demonstrated their success in preparing Pd@Pt core@shell NPs via epitaxial growth and subsequently evolving into PdH_0.43_@Pt NPs [109]. As shown in Fig. 6e–h, HAADF-STEM and EDX elemental mapping images validated the formation of Pd@Pt NPs and PdH_0.43_@Pt NPs. After hydrogen intercalation, the Pd core could convert to PdH_0.43_, and the core lattice expansion led to the lattice enlargement of the Pt shell.

**Fig. 7:**
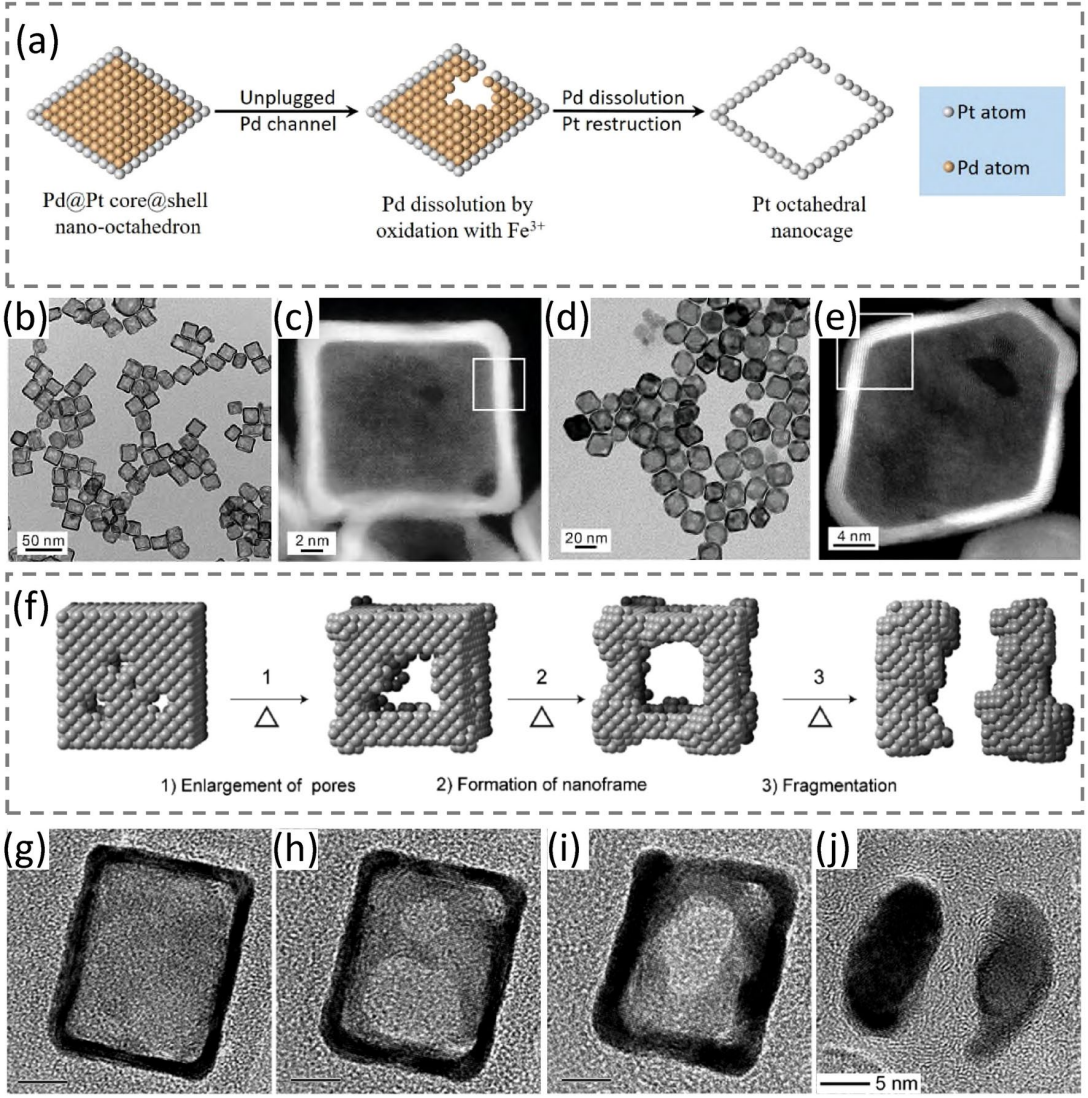
Schematic illustration and TEM images of Pt-based nanocages. **a** Schematic illustration of Pd atoms dissolution from a Pd@Pt nano-octahedron, generating a Pt octahedral nanocage. **b, c** TEM and HAADF-STEM images of cubic nanocages, respectively. **d, e** TEM and HAADF-STEM images of octahedral nanocages, respectively. **b**–**e**: Reproduced with permission from Ref. [89]. Copyright 2020 American Chemical Society. **f** Schematic illustration of the process by which a Pt nanocage breaks down under thermal stress. **g–j** In situ HRTEM images, showing the evolution of a Pt cubic nanocage (**g)** before and **h–j** after different stages of heating treatment: **h** 400 °C for 20 min, **i** 400 °C for 60 min, and **j** 500 °C for 30 min. All the data bars represent 5 nm. Reproduced with permission from Ref. [110] and modified. Copyright 2018 Wiley-VCH

The Xia group further demonstrated their synthesis of nanocages (Fig. 7) by depositing a few atomic layers of Pt as conformal shells on facet-defined Pd NCs while the Pd template was subsequently etched away. The schematic representation of the dissolution of Pd and formation of Pt cubic nanocages is shown in Fig. 7a. TEM and HAADF-STEM images verified the formation of Pt cubic nanocages terminated with {100} facets (Fig. 7b–c) and Pt octahedral nanocages bounded with {111} facets (Fig. 7d–e), respectively. Importantly, the thermal stability of the Pt-based nanocages was investigated using in situ electron microscopy, revealing a thermodynamically driven process that transforms nanocages into NFs upon the heating process and demonstrating the possibility to enhance their thermal stability by controlling the surface morphology and location of the pores. Figure 7f illustrates the nanocage evolution, including (1) pre-existing pores on the surface of the nanocage are enlarged; (2) the edges and corners are thickened due to the migration of atoms away from the pore-decorated side faces; (3) the resultant formation of an NF with open side faces. Figure 7g–j are in situ HRTEM images, demonstrating the morphology change of the nanocage upon different heating conditions. The morphology development of the nanocage confirmed the schematic assumption displayed in Fig. 7f.

### Morphology Control of Pt-Based Intermetallic Compounds

**Fig. 8:**
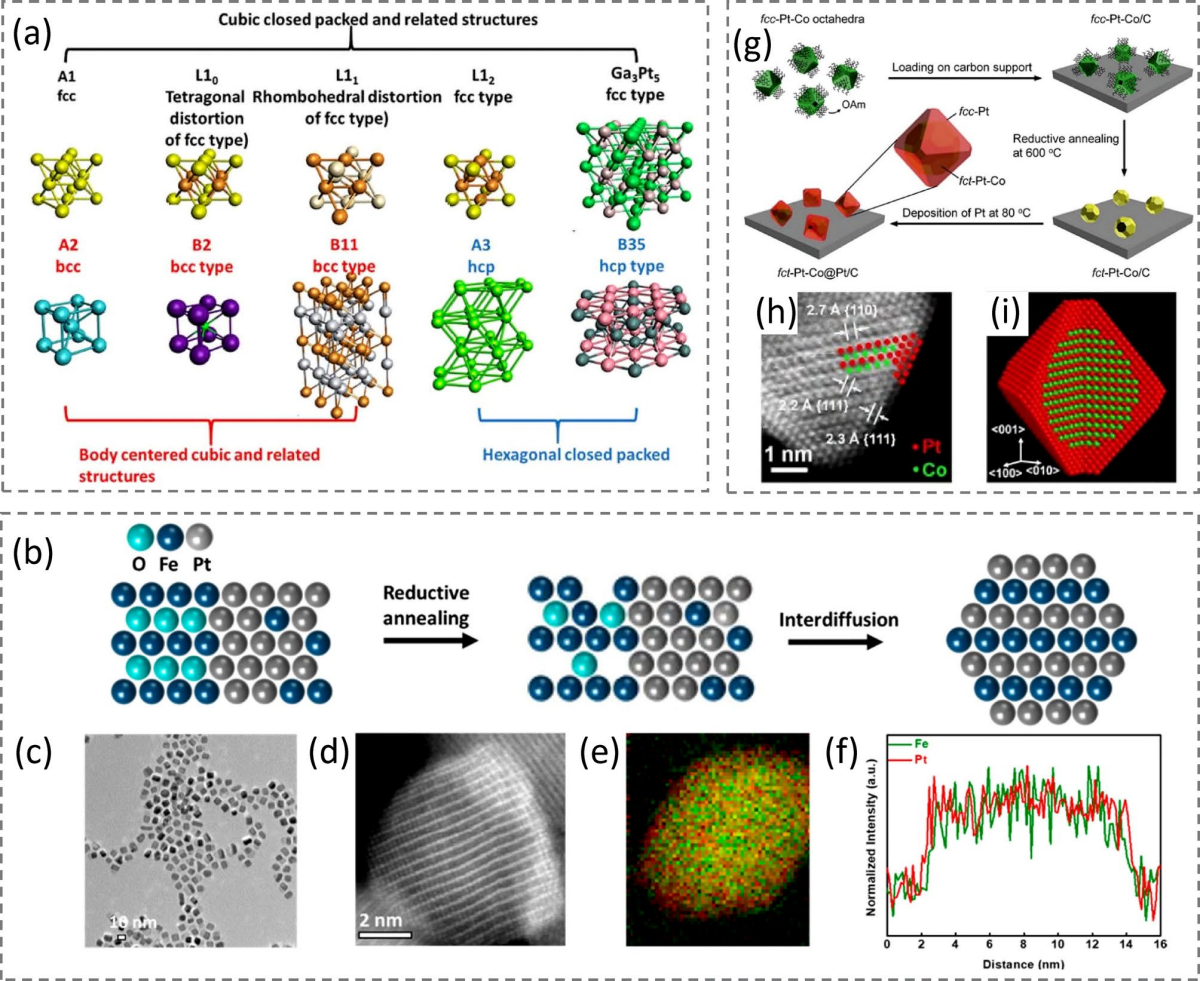
**a** Illustration of some simple alloy structures and their corresponding intermetallic structures. **b** Schematic of structural conversion from dumbbell-like Fe_3_O_4_–PtFe NPs to L1_0_–PtFe via reductive annealing.** c** TEM image of dumbbell-like Fe_3_O_4_–PtFe alloy nanoparticles. **d–f** HAADF-STEM image, elemental map, and line-scanning profile of the converted L1_0_–PtFe nanoparticle, respectively. Reproduced with permission from Ref. [11] and modified. Copyright 2009 American Chemical Society. **g** Schematic illustration of the three major steps involved in a typical synthesis of *fct*-Pt-Co@Pt/C catalyst. **h** Atomic-resolution STEM image of the Pt–Co@Pt octahedral nanocrystal along the [1$\overline{1 }$0] direction.** i** Schematic of this nanocrystal featuring an intermetallic core, a Pt shell of about three atomic layers thick, and (111) facets. The red- and green-colored atoms correspond to Pt and Co, respectively. Reproduced with permission from Ref. [114]. Copyright 2021 American Chemical Society

A single-phase solid solution consisting of mixed Pt and other components generally refers to an alloy, in which the atoms are randomly distributed across the lattice. Therefore, such a solid solution structure is also called a random alloy. A structurally ordered alloy is usually categorized as an intermetallic compound that has strict stoichiometry, an atomically ordered arrangement, and a well-defined atom-binding environment [2, 111]. Some simple random alloys and their corresponding intermetallic crystal structures are displayed in Fig. 8a. As a transition example between the alloys and intermetallics, the conversion from alloy Pt-Fe in dumbbell-like Fe_3_O_4_–PtFe NPs to intermetallic Pt-Fe via reductive annealing/interdiffusion is schematically illustrated in Fig. 8b. The A1–PtFe (*fcc* phase alloy) was first synthesized at 200 °C using a similar approach described in Sect. 3.2 [36], followed by the overgrowth of Fe_3_O_4_ from the oxidation of excess amount of Fe(CO)_5_, forming dumbbell-like Fe_3_O_4_–PtFe NPs [11]. During the next reductive annealing stage at 700 °C for 6 h in 5% H_2_/Ar, vacancies are created from the Fe_3_O_4_ due to the removal of oxygen and this promotes the full conversion of intermetallic L1_0_–PtFe from the dumbbell-like Fe_3_O_4_–PtFe alloy NPs through the diffusion of metallic atoms to the generated oxygen vacancies [112, 113]. The HAADF-STEM image, elemental map, and line-scanning profile confirm the formation of L1_0_–PtFe NP (Fig. 8c–f) [112].

Compared to the alloy phase of Pt-based NCs that are usually yielded at relatively low synthesis temperatures, Pt-based intermetallic counterparts are typically generated at higher annealing temperatures through a disorder-to-order transition process. This thermal annealing protocol is more prevalent in producing Pt-based intermetallic NCs with a structure-ordered phase, and we have reviewed these systems previously [2, 3]. Interestingly, there are still some exceptions, such as the Pt–Sn, Pt–Pb, Pt–Bi, and Pt–Ga intermetallic compounds, which can be directly achieved in a hot colloidal solution. The details are outlined in Table 4. For instance, monodisperse intermetallic Pt_3_Sn NCbs were directly synthesized by co-reducing Pt and Sn precursors at 240 °C in a colloidal system using a hot injection method [12]. The ordering degree of these Pt_3_Sn NCbs could be further reduced by thermal treatment at 200 and 250 °C (around the phase transition temperature of Pt_3_Sn) in air, while their shape and size were preserved.

**Table 4:** Summary of some Pt-based intermetallic NCs synthesized in colloidal solutions

	Morphology	Preparation condition	References
Pt–Bi	2D nanoplate	Pt(acac)_2_+ Bi(Ac)_3_+ AA + NH_4_Br at 160 °C	[118]
Pt–Ga	–	Pt(acac)_2_ + GaCl_3_ at 300 °C	[119]
Pt–Pb	2D nanoplate nanorod	Pt(acac)_2_ + Pb(acac)_2_ + L-AA at 160 °C Pt(acac)_2_+ Pb(acac)_2_ + 1-adamantanecarboxylic acid + hexadecanethiol + tert-butylamine borane complex at 180 °C	[120, 121]
Pt–Sn	Cube	PtCl_2_+ SnCl_2_ ·2H_2_O + tetradecanediol + dodecylamine at 240 °C	[12]
	Concave nanocube	Pt(acac)_2_+ SnCl_2_ ·2H_2_O + PVP at 180 °C	[122]
Pt–Co	Octahedron	Ordered Pt-Co NCs + K_2_PtCl_4_+ citric acid at 80 °C	[114]

*L-AA* L-ascorbic acid, *PVP* poly(vinyl pyrrolidone), acac acetylacetonate, *Ac* acetate

Unfortunately, the phase transition temperatures of most Pt-alloys, such as Pt–Co, Pt–Ni, and Pt–Fe, are much higher than 250 °C, requiring a high annealing temperature (> 500 °C) to allow the disorder-to-order transition. This impedes morphology preservation during the thermal treatment due to the lack of capping ligands or protecting templates to stabilize the high surface energy atoms at the high annealing temperature, leading to nano-polyhedra or nanospheres after the intermetallic conversion [112, 115, 116]. For example, the surface evolution and composition-dependent ordering transformation processes of Pt–Fe NCbs have been in situ observed on the single-particle level using electron microscopy [117]. During vacuum annealing, this ordering occurs via either “surface nucleation and growth” or “interface” mechanism, resulting in a core–shell configuration. Meanwhile, the cubic morphology was converted to a spherical shape, instead of facet preservation. To tackle this difficulty, the Xia group recently employed an epitaxial growth strategy and developed Pt–Co@Pt octahedral NPs that feature intermetallic, face-centered tetragonal (*fct*) Pt–Co cores and ultrathin Pt-shells terminated with {111} facets [114]. As illustrated in Fig. 8g–i, the disordered Pt–Co alloy NCs synthesized from a typical colloidal system were first loaded on a carbon support, followed by reductive annealing at 600 °C under H_2_/Ar. This resulted in a transition from the *fcc* Pt–Co alloy to *fct* Pt–Co intermetallic NCs. Meanwhile, the surface of each NC was also coated with a conformal and ultrathin shell of Pt, generating *fct* Pt–Co@Pt octahedral NCs. The atomic-resolution STEM image indicates that the resultant NC was composed of a highly ordered intermetallic core with alternating Pt and Co atomic layers and a smooth Pt shell of three or four atomic layers in thickness (Fig. 8h). Figure 8i shows a 3D model of such an NC with the layer-by-layer atomic arrangement.

## Case Study: Electrocatalytic Applications of Pt-Based Nanocrystals

Prior to the characterization and catalytic evaluation, NCs synthesized from the colloidal system need to be isolated from the reaction media by centrifugation, followed by dispersion into a non-polar solvent and precipitation (via centrifugation) with polar solvent for several cycles to remove the byproducts and majority of the surfactants. It is believed that, in such a case, the NCs are still stabilized against agglomeration by a small amount of adsorbed surfactants, which could seriously affect the property assessment. If necessary, the NCs can be further cleaned after they are loaded on substrate or support (to avoid their accumulation). There are several additional methods for further removing the surfactants capped on the NCs, including (1) electrochemical activation [123, 124], (2) solvent washing/ligand exchange process [125–127], and (3) thermal annealing treatment [128–130]. A straightforward procedure for removing surfactants (*i.e.*, PVP, OAms, OA) from the obtained Pt-based NCs can be conducted by electrochemical potential cycling in acid (e.g*.*, 0.5 M H_2_SO_4_) or alkaline media (e.g*.*, 0.5 M NaOH solution) until achieving a stable cyclic voltammogram. It was reported that the electrochemical potential cycling in acidic media with an appropriate upper potential limit can remove most PVP, OAm, and OA on Pt NCbs but less effectively compared with the alkaline media [123], especially for the removal of PVP. For the removal of some organic acids (*e.g.*, OA or acetic acid), the use of NaOH-ethanol solution is a good choice [127, 131]. In addition, it was also reported that annealing the NCs in air, after loading onto carbon black, at an optimized temperature for a certain period could have the NC surface cleaned effectively without altering the size and morphology [129, 130]. Compared to oxidative annealing or UV-ozone processing [132], plasma treatment is another attractive method to remove the organic species from NCs [133]. In order to maximize the removal of organic species without changing NC in size, morphology, and composition, the aforementioned cleaning protocols are sometimes combined prior to the assessment of catalytic performance.

Heterogeneous catalysis usually involves diffusion of reactants, adsorption on the interface of different phases, reactions, and desorption of products. It has been over a century since Paul Sabatier proposed the Sabatier principle when he studied the hydrogenation and dehydrogenation of catalysts [2]. This general principle stated that the *BE* should be balanced properly to optimize its catalytic performance (*i.e.,* reactivity and selectivity). In recent years, modern electronic structure theory allows the quantitative analysis of the “reactivity on the catalyst surface.” To uncover the nature of the active sites on metal catalysts, the linear Brønsted-Evans-Polanyi (BEP) relationship was postulated by Bligaard and Nørskov [134, 135]. The BEP lines provide a protocol to break down the structure sensitivity into independent geometrical and electronic effects. Thanks to the fast development of computation methods/models in quantum chemistry, the density functional theory (DFT) is an electronic density function-based calculation, instead of wave-function-based calculation (the Ab initio method), and now has been a popular tool to simulate the surface electronic structure and investigate the surfaces reaction mechanisms [119, 135–137]. With the systematic combination between experimental characterization techniques and theoretical quantum chemistry, an in-depth understanding of the reactant interaction on heterogeneous catalysts becomes possible, facilitating the reaction improvement through optimizing the catalysts. Based on these perspectives, in this section, we outline some recent studies on the electrochemical applications of Pt-based NCs by focusing on ORR and small molecular (*e.g.*, methanol and formic acid) oxidation reactions, and highlight how the morphology of NCs affect their electronic surface density and catalytic performance.

### Oxygen Reduction Reaction

Pt-alloy NCs are considered the most promising candidates as cathode catalysts to promote ORR kinetics. To minimize the *BE* between oxygen species and Pt active sites which is generally too strong, there have been substantial efforts in developing Pt-based bimetallic NCs and optimizing their electronic structures by tuning the Pt fraction toward ORR of proton-exchange membrane fuel cell (PEMFC) [138–140]. Typically, three strategies have been used to boost the ORR performance: (1) incorporation of 3*d* transition metals into Pt lattice with size, composition, and morphology control, (2) transformation of the most ORR-promising crystal facets from single crystal to NCs, and (3) development of core–shell NPs where the Pt or Pt-alloy component is designed as the shell [1, 2, 4, 6, 29, 31, 141, 142].

**Fig. 9:**
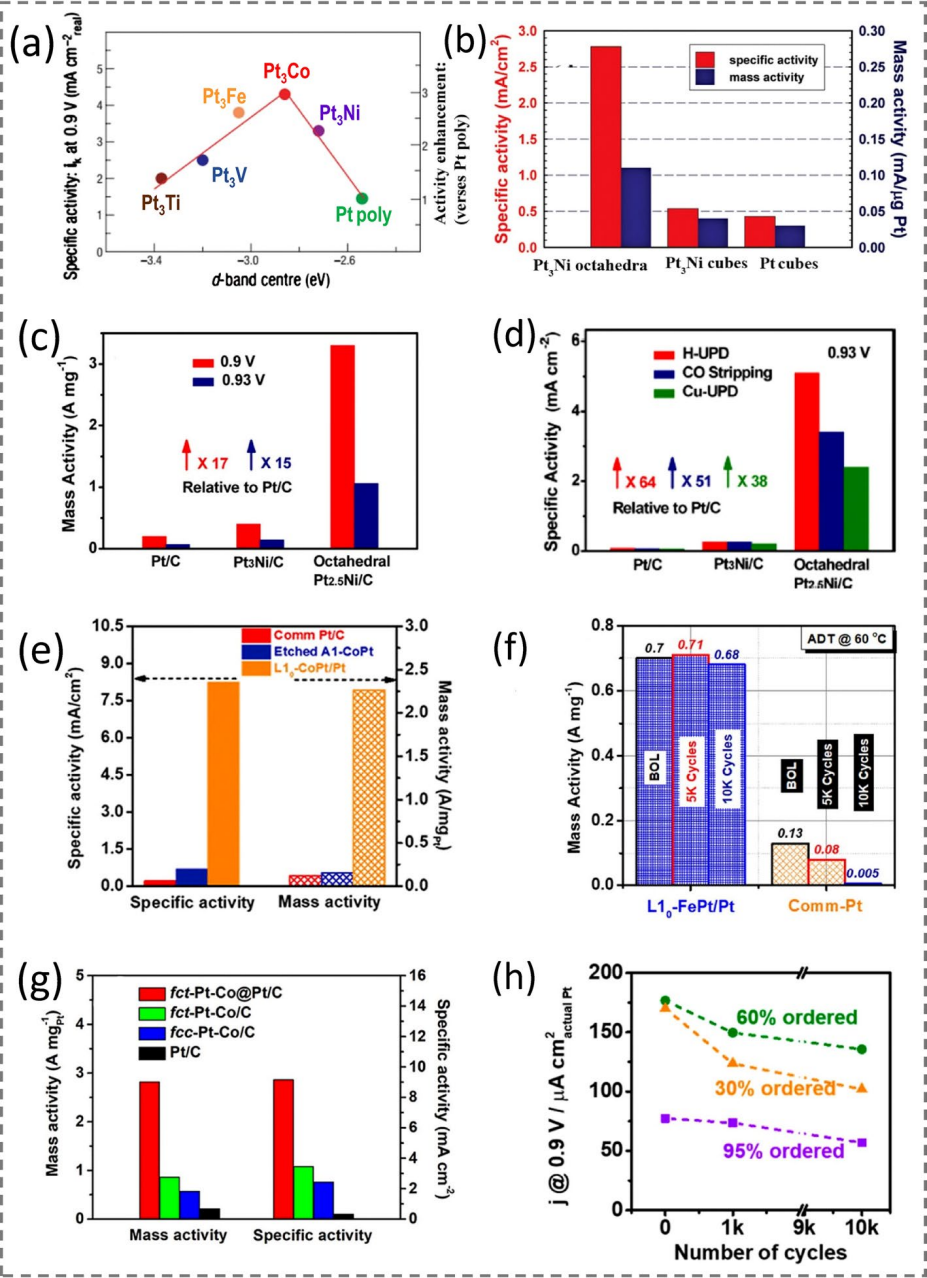
**a** Relationships between experimentally measured specific activity for the ORR on Pt_3_M surfaces in 0.1 M HClO_4_ at 333 K and the *d*-band center position for the Pt-skin surface. Reproduced with permission from Ref. [143] and modified. Copyright 2019 Wiley-VCH. **b** Comparison of the ORR activities among the three types of nanocatalysts (evaluated at 0.9 V *vs.* RHE at 295 K). Reproduced with permission from Ref. [17]. Copyright 2010 American Chemical Society. **c, d** Comparison of Pt mass activity and specific activity of Pt/C (TKK), Pt_3_Ni/C (TKK), and octahedral Pt_2.5_Ni/C, respectively. Reproduced with permission from Ref. [22]. Copyright 2013 American Chemical Society. **e** Specific activity and mass activity of comm. Pt/C, etched A1–CoPt, and L1_0_–CoPt/Pt (measured at 0.9 V *vs.* RHE). Reproduced with permission from Ref. [116]. Copyright 2018, Elsevier. **f** Mass activity of L1_0_–FePt tested at the beginning of life (BOL) and after the ADT test (5,000 cycles at 60 °C in oxygen-saturated 0.1 M HClO_4_). Reproduced with permission from Ref. [112]. Copyright 2018 American Chemical Society. **g** Mass activity and specific activity of different catalysts at 0.9 V *vs.* RHE based on the Pt mass loading. Reproduced with permission from Ref. [114]. Copyright 2021 American Chemical Society. **h** Mass kinetic current densities normalized by the ECSA that was measured after each ADT cycle of the Pt_3_Sn nanocubes with different degrees of ordering at 0.9 V *vs.* RHE. Reproduced with permission from Ref. [12]. Copyright 2020 American Chemical Society

In 2007, the Markovic group stated that Pt_3_M (M = Ti, V, Fe, Co, Ni) surface exhibits “volcano-type” behavior when they studied the relationship between the *d*-band center and ORR activity (Fig. 9a), indicating high ORR activity on Pt_3_Ni and Pt_3_Co surfaces [14]. Importantly, by showing the enhanced ORR specific activity (SA) on Pt_3_M surfaces corresponding to their average *d*-band center positions, Fig. 9a implies that most Pt-alloys possess favorable electronic structure modulation toward ORR compared to pristine Pt. They also determined that extended single crystal surfaces of Pt_3_Ni(111) exhibited an enhanced ORR SA that is tenfold higher than that of Pt(111) and 90-fold higher than that of state-of-the-art Pt/C catalysts [13]. The superior ORR activity indicates an unusual electronic structure (*i.e.*, *d*-band center position) and surface atom arrangement. Compared with the pure Pt(111), the *d*-band center for Pt_3_Ni(111) shifts downward about $|\Delta {d}_{111}|\hspace{0.17em}$=  ~ 0.34 eV. The facet-dependent activity on extended single crystal surfaces (100) and (110) was also studied. Since Pt_3_Ni(111) single crystal surface exhibits the highest ORR activity, a W(CO)_6_-based synthesis protocol was developed later on (see Sect. 3.2) and the Pt_3_Ni(111) surface was “transferred” to nanophase by successfully presenting monodisperse Pt_3_Ni nano-octahedra which are exclusively terminated with {111} [17]. As expected, the Pt_3_Ni nano-octahedra exhibit the highest ORR performance (both in mass activity (MA) and SA at 0.9 V) in comparison with {100}-bounded Pt_3_Ni NCbs and Pt NCbs in a similar size (Fig. 9b). This ORR performance was significantly improved shortly after the Pt–Ni nano-octahedra were post-treated with acetic acid [22], reaching MA value of ~ 3.3 A mg_Pt_^−1^ which is 17 times as high as that of state-of-the-art Pt/C catalyst (Fig. 9c) with 64-fold SA value as high as that of Pt/C catalyst (Fig. 9d).

Seed-mediated epitaxial growth has been used to systematically develop shape-controlled core@shell Pt-alloy NCs. For example, Pt was deposited on shape-controlled Pd on atomic scale NCs to generate Pd@Pt core@shell nanostructures in various morphologies (cubes, concave cubes, octahedra, icosahedra, and concave decahedra) while the thermodynamic and kinetic parameters were well-controlled [29, 46, 97, 98, 102]. In the process of Pd@Pt concave NDs, Pt atoms prefer to stay at the vertices and edges/ridges of the Pd decahedral seeds owing to the twin boundaries and twin defects over the surface of Pd cores. This is different from the other shapes of Pd cores, such as NCb, nano-octahedron, and NI. As a result, the Pd@Pt concave NDs with 2 atomic Pt layers exhibit the highest ORR MA (1.60 A mg_Pt_^−1^) compared to Pd@Pt NCs in other shapes [100].

An elemental dissolution (or leaching) problem always exists on Pt-alloy or core@shell NCs when they are used as ORR electrocatalysts, due to the different activities between Pt and the other element. One of the solutions to mitigate this issue is to adopt Pt-based intermetallic NCs. In such a case, the mobilities and leaching rate of the second metal atoms could be significantly reduced due to their stronger binding with Pt atoms. This has been demonstrated using PtCo and PtFe systems, showing intermetallic L1_0_–PtCo and L1_0_–PtFe NCs possess significantly enhanced compositional stability and ORR durability, as well as improved MAs (Fig. 9e–f) [112, 116]. However, as mentioned in Sect. 3.4, the disordered-to-ordered transition for most Pt-based systems requires high-temperature annealing (> 500 °C). It is a challenge to preserve the NC morphology during this thermal treatment process.

Two strategies have been reported to produce Pt-based intermetallic NCs with exclusive facets. One is the epitaxial growth on an intermetallic core, and the other is a low-temperature disordered-to-ordered transition. For example, it has been demonstrated that intermetallic *fct* Pt–Co@Pt nano-octahedra could be produced by epitaxially growing Pt layers on intermetallic *fct* Pt–Co truncated octahedral cores that were pre-converted from as-synthesized *fcc* Pt–Co nano-octahedra at 600 °C for 4 h (Fig. 8g–i) [114]. The resultant intermetallic core@shell NCs show over the fivefold enhancement of MA (2.82 A mg_Pt_^−1^) toward ORR compared to the *fcc* Pt–Co octahedral alloy NCs (0.57 A mg_Pt_^−1^), and also exhibit remarkable durability with activity largely retained even after 30,000 cycles of accelerated durability test (Fig. 9g). As mentioned in Sect. 3.4, some Pt-alloys, such as Pt_3_Sn, possess low disordered-to-ordered transition temperature, making the morphology-preserved annealing of NCs possible. For example, it was recently reported that the ordering degree of Pt_3_Sn NCbs could be altered by varying the annealing temperatures (200 and 250 °C) in air [12]. The high ordering degree of Pt_3_Sn NCbs results in better electrocatalyst stability against the dissolution of the elements during the electrocatalysis, whereas the low ordering degree seems to offer higher catalytic activity as shown in Fig. 9h.

### Small Molecule Electro-Oxidation Reaction

#### Oxidation Reaction in Acidic Media

**Fig. 10:**
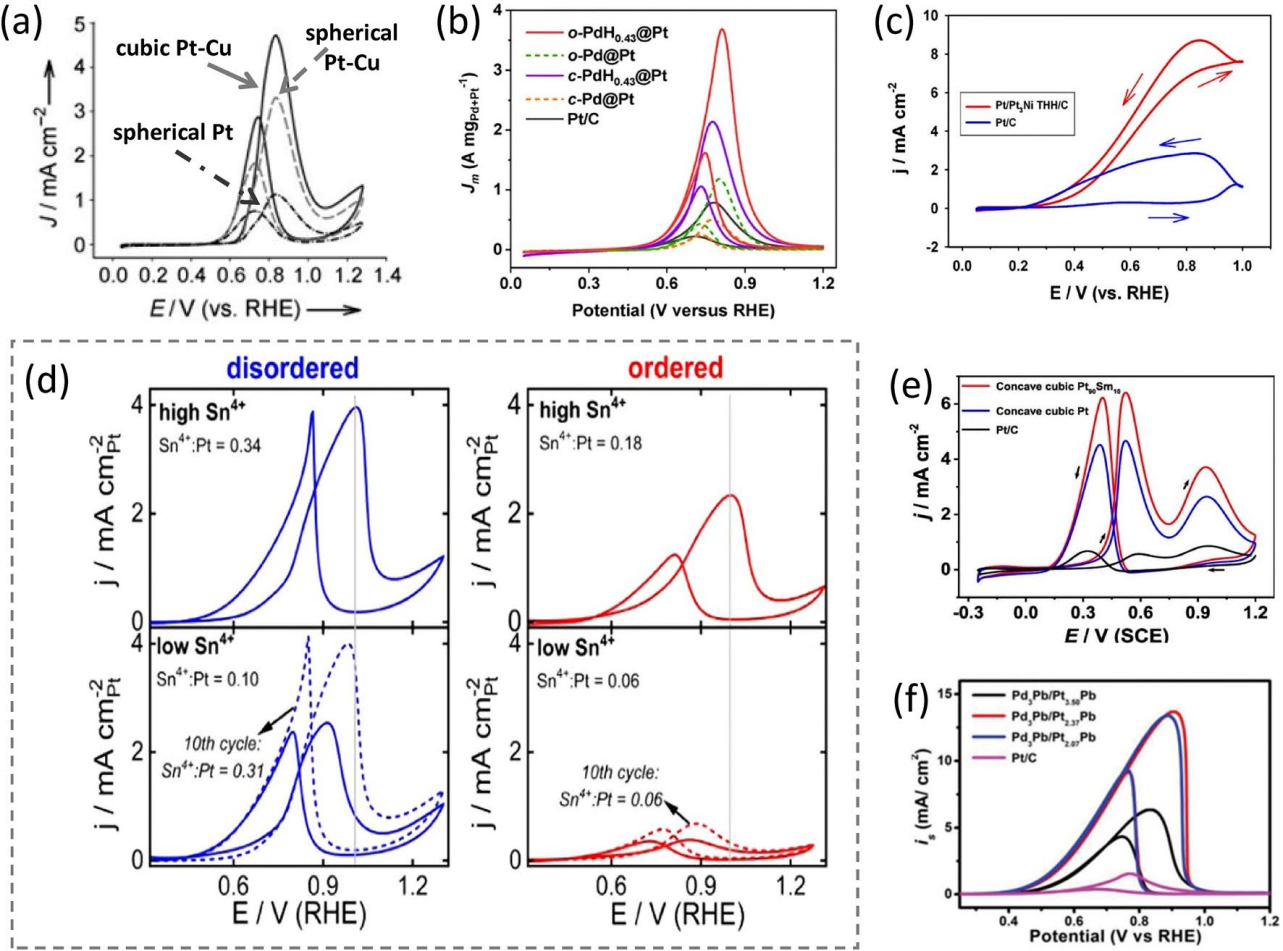
**a** CV profiles of methanol oxidation on Pt–Cu nanocubes, Pt–Cu nanospheres, and Pt nanospheres in 0.1 M HClO_4_ + 1 .0 M methanol (scan rate: 20 mV s^−1^). Reproduced with permission from Ref. [37]. Copyright 2009 Wiley-VCH. **b** CV curves of methanol oxidation of *c*-Pd@Pt, *c*-PdH_0.43_@Pt, *o*-Pd@Pt, *o*-PdH_0.43_@Pt, and commercial Pt/C electrocatalysts measured in 1.0 M KOH containing 1.0 M methanol, where the *c* stands for the cubic, and *o *stands for the octahedral. Reproduced with permission from Ref. [109]. Copyright 2021 American Chemical Society. **c** Comparison of electrocatalytic activity per unit Pt surface area between Pt_3_Ni tetrahexahedra/C and Pt/C (CVs in 0.1 M HClO_4_ + 0.5 M HCOOH at a scan rate of 0.1 V s^−1^). Reproduced with permission from Ref. [67] and modified. Copyright 2017 American Chemical Society. **d** Cyclic voltammograms in 0.1 M HClO_4_ + 1 M methanol of disordered high Sn^4+^, disordered low Sn^4+^, ordered high Sn^4+^, and ordered low Sn^4+^ Pt_3_Sn nanocubes. Reproduced with permission from Ref. [147]. Copyright 2021 American Chemical Society. **e** CV (50 mV s^−1^) measured at 0.45 V (*vs.* SCE) of ethanol oxidation in 0.1 M ethanol + 0.1 M HClO_4_. Reproduced with permission from Ref. [148]. Copyright 2019 American Chemical Society. **f** CV of different catalysts measures using the solution containing 1 M KOH and 1 M MeOH at a scan rate of 50 mV s^−1^ for the methanol oxidation reaction. Reproduced with permission from Ref. [149]. Copyright 2019 Wiley-VCH

Except for hydrogen oxidation reaction (HOR), small organic molecules, such as methanol, formic acid, and ethanol, can also be used as the anode fuels in PEMFCs. Consequently, the anodic electrocatalysts play a critical role in facilitating these reactions. Due to the "suitable" electronic structure, Pt-based NCs are still one class of the most promising nanocatalysts at the anode of PEMFCs. The Fang group produced several classes of Pt-based NCs and investigated their electrochemical oxidation performances [17, 30, 37, 67, 108, 144]. These anodic catalysts include shape-controlled Pt–Cu NCs, Pt–Ni THH NCs, and CuNi@Pt–Cu core@shell nano-octahedra. For instance, they have pioneered the electro-oxidation catalysis of Pt–Cu NCbs toward methanol oxidation reaction (MOR) in 0.1 M HClO_4_ + 1.0 M methanol [37]. As shown in Fig. 10a, Pt–Cu NCbs exhibited the highest current density (4.7 mA cm^−2^) toward MOR in comparison with Pt–Cu nanospheres (3.1 mA cm^−2^) and Pt nanospheres (1.1 mA cm^−2^). Shortly, they reported the formic acid oxidation reaction (FAOR) performance of Pt–Cu nano-octahedra, NCbs, and Pt NCbs in 0.1 M HClO_4_ + 0.5 M HCOOH. The highest current density was observed on Pt–Cu NCbs in the range of 0.6 ~ 0.9 V *vs.* reversible hydrogen electrode (RHE), whereas the Pt–Cu nano-octahedra showed the lowest current density for FAOR [40]. These catalytic behaviors toward FAOR indicate that the Pt–Cu NCbs are more favorable for FAOR than pure Pt NCbs due to the weaker *BE* between CO and Pt–Cu NCbs, which is caused by the introduction of Cu into the Pt lattice. Furthermore, the Pt–Cu catalyst activity for FAOR was facet-dependent, that is, Pt–Cu(100) facet was more active than Pt–Cu(111) facet. Recently, morphology-controlled CuNi@Pt–Cu octahedral NPs were successfully developed [108] (see Sect. 3.3), and these NPs supported on active carbon showed superior MA (0.99 A mg_Pt_^−1^) and SA (7.49 mA cm^−2^) toward electrochemical MOR compared with their counterparts, carbon-supported CuNi@Pt–Cu nanospheres (MA = 0.66 A mg_Pt_^−1^, SA = 5.57 mA cm^−2^) and benchmark Pt/C catalyst (MA = 0.23 A mg_Pt_^−1^, SA = 1.30 mA cm^−2^), indicating that the (111)-profiled Pt–Cu shell facets and lattice mismatch between the CuNi core and the Pt–Cu shell contribute to the improvement of this catalytic performance. As shown in Fig. 10b, hydrogen intercalation of core@shell Pd@Pt NCs can further expand the Pt-shell lattice, leading to a significantly enhanced performance of MOR on both Pt(100) and the Pt(111) [109]. These results suggest that the lattice expansion could endow both (100) and (111) facets of Pt with improved catalytic activities.

Recent studies also revealed that high-index facets of noble metal (such as Pt) NCs could improve the electrocatalytic activity toward small molecule oxidation reactions since these facets expose abundant active sites with low CNs of Pt-like atoms [79, 145]. One of the examples is that Pt_3_Ni THH NFs/C that were derived from {730}-terminated PtNi_4_ THH NCs through a Mond process (see Sect. 3.2) [67] exhibited higher FAOR SA (~ 8.5 mA cm^−2^) compared to the benchmark Pt/C catalyst as shown in Fig. 10c, indicating they have much better tolerance on CO poising due to the low CNs of Pt atoms on frames as well the compressive strain from surface Pt-skin. It was also reported that PtCo-excavated rhombic dodecahedral (ERD) NCs showcased remarkably higher activity toward MOR, benefiting from the excavated structure and the alloying feature [146].

Pt–Sn NC is a special system, which is used to study the facet and disorder/order effect on electrochemical catalysis due to its low phase transition temperature between random alloy and intermetallic compound (see Sect. 3.4). In addition to the influence on ORR performance (see Sect. 4.1), it was reported that the ordering degree of Pt_3_Sn NCbs also impacts MOR performance [147]. Particularly, the Sn atoms in 60% ordered Pt_3_Sn NCbs can be electrochemically oxidized to Sn^4+^, whereas those in 95% ordered Pt_3_Sn NCbs are more resistant to this oxidation. As the resultant Sn^4+^ ions on the catalyst surface would create more active sites on disordered Pt_3_Sn NCbs than on ordered NCbs, the 60% ordered NCbs exhibit 5.6-fold higher MOR activity than the 95% ordered NCbs as illustrated in Fig. 10d, showing the significant impact of the atom arrangement on the catalytic performance.

As shown in Fig. 10e, Pt–Sm alloy concave NCbs enclosed with high-index facets {310} are another example of enhancing electro-oxidation activity toward ethanol oxidation reaction (EOR) compared to their counterparts, Pt concave NCbs and benchmark Pt/C. This superior performance is attributed to the synergy of steric and electronic effects from the high-index faceted alloy [148]. Pd_3_Pb/Pt_n_Pb NCbs with tunable Pt composition also exhibit their substantial electro-oxidation performance toward MOR [149]. The hexagonal-structured PtPb intermetallics in the Pd_3_Pb/Pt_2.37_Pb catalyst show the highest performance compared to commercial Pt/C and other studied samples (Fig. 10f).

Unlike MOR and FAOR which do not involve a breaking of the C–C bond, the EOR is often incomplete due to the difficulty in the C–C bond cleavage. EOR normally results in several types of byproducts other than the 12-electron oxidation product of CO_2_. It was demonstrated that PtNiRh ternary alloy NPs show highly promising performance toward the electrochemical EOR [150]. Furthermore, PtNiRh nano-octahedra (*fcc* alloy) exhibited excellent activity and unachieved low onset potentials (as low as 0.1 V *vs.* RHE) toward EOR, showing high selectivity to completely oxidize ethanol to CO_2_ [151]. An additional mechanistic study using in situ FTIR reveals that a low Rh content that is well-distributed on surface sites of the ternary {111} facets favors the C_1_ pathway of EOR, whereas the highest Rh content formed as Rh shell supports the C_2_ pathway. Recently, single-atom catalysts (SACs) have been drawing great attention as a new type of electrocatalyst. In SACs, a trace amount of metal catalyst is atomically dispersed on the support and the surfaces of metallic atoms are sufficiently utilized [152, 153]. For instance, unalloyed single atomic Rh decorated on the Pt NCbs served as a new type of SAC catalyst, demonstrating a complete EOR efficiently [154]. Although the onsite potential (0.35 V *vs.* RHE) is not as low as that of aforementioned PtNiRh octahedral nanocatalysts (0.10 V *vs.* RHE), the Rh SAC sites and Pt NCbs can synergistically promote the C–C bond cleavage and the removal of the *CO intermediates. This study offers a unique single-atom strategy on shape-controlled nanocatalysts to tune the reactivity of other complicated catalytic reactions.

#### Oxidation Reaction in Alkaline Media

In contrast to the acidic electrolytes, small molecule oxidations (including HOR) in alkaline media are more sluggish, and the reaction rates are 2–3 orders of magnitude slower than those in acid [155, 156]. Therefore, it is essential to develop highly efficient electrocatalysts for HOR, MOR, and EOR in alkaline media, intrigued by the anion exchange membrane fuel cells (AEMFCs). It has been reported that in alkaline media, the H_2_ and/or H_2_O/OH^−^ species dominate the HOR reaction steps (Heyrovsky step and Volmer step). Since it is well-known that Pt is active for H_2_ dissociation, incorporation of Pt with some exophilic components such as Ni, Ru, or Ir would optimize the balance between the adsorption/dissociation of H_2_ and the adsorption of hydroxyl species (OH_ad_) on the active sites, yield several-fold higher HOR activities compared to the state-of-the-art Pt catalysts in alkaline environments [157, 158]. The HOR activity on low-index facets of single crystal Pt in alkaline media was reported as Pt(111) < Pt(100) < Pt(110) [159]. By introducing Ni atoms into the Pt lattice, it was determined that the HOR activity on single crystal Pt(111) in 0.1 M KOH is affected by the Ni content, giving the following order: Pt_3_Ni(111) < PtNi(111) < PtNi_surface_(111) < PtNi_3_(111). Besides, Pt NCs with high-index facets possess abundant atomic steps and kinks with low-CN sites and can serve as a promising electrocatalyst to adsorb reaction species/intermediates and break C–C bonds subsequently [160]. Although it was reported that the step atoms on high-index facets of Pt single crystal were the active sites for HOR in an acid [161], little attention is paid to the high-index facets of Pt or Pt-based NCs toward HOR in alkaline media, and further investigations are expected.

## Conclusions and Perspective

Various types of shape-controlled Pt-binary NCs have been developed. Studies have also established different relationships between their structure, including the morphology, size, composition, as well as crystallographic phase, and remarkable catalytic properties. The associated syntheses can be precisely controlled and optimized through in situ implementation and/or post-treatment, whereas the properties can be achieved and tuned by the impact of the ligand effect, ensemble effect, and strain effect. Among these, Pt-based NCs with shape control have been well-recognized as a typical class of promising catalysts for electrochemical applications, and strategies for material preparation have been progressively developed.

Despite the significant achievement in colloidal synthesis, there is still a challenge in synthetic protocol development and corresponding mechanistic understanding. Nucleation and subsequent growth of NCs associated with facet evolution are two critical stages in a colloidal system for the preparation of Pt-based NCs. By employing the liquid cell TEM technique, it is now possible to monitor the growth/evolution and in situ catalysis of NCs, offering a direct avenue to observe the facet development during the seed growth as well as the morphology variation within the electrocatalysis process. However, only limited cases, such as Pt NCbs [49], Pt–Fe nanorods [53], Pd@Pt core@shell NCbs [162], Pd@Au and Cu@Au core–shell NCbs [163], have been well investigated. Extensive efforts are needed to uncover the insight into the morphology evolution for other metallic NC systems, especially those with high-index facets. It is worth mentioning that the intensity of the electron beam should be appropriately adjusted when observing the facet development using an in situ TEM because with a high dose, electron irradiation could kinetically interfere with the evolution of the sample [54, 164, 165]. Moreover, the morphology control could be lost during electrocatalytic cycling due to the possible aggregation or shape degradation, resulting in the decay of high reactivities of the shape-controlled catalysts. Therefore, understanding this structural degradation is critical [166, 167]. For example, observation at an aberration-corrected STEM indicated that during the electrochemical process, the dissolution preferentially occurred at the steps and corners of Pt–Ni NCs and the redeposition process often happened on Pt–Ni {111} planes, leading to their disappearance and an anisotropic growth of smaller particles [167]. These studies suggest that maintaining structural stability in catalysis remains a challenge, and that further in-depth investigations are necessary.

Concerning the synthesis of Pt-based intermetallic NCs with morphology control, limited examples were published compared to cases of Pt-based random alloy NCs. Three strategies are proposed to tackle this challenge: First, to select the Pt-based systems with a relatively low disordered-to-ordered transition temperature, which should typically be lower than the synthesis temperature (*i.e.*, Pt–Sn NCbs as discussed in Sect. 3.4); second, to adopt an epitaxial growth pathway on a structure-ordered NC as the growth seed, followed by a morphology-controlled “coating” direct and develop the exposed facets as desired in a colloidal system (*e.g.*, the demonstrated intermetallic Pt–Co@Pt nano-octahedra [114]); third, to develop novel “capping ligands” that could tolerate a higher reaction temperature tolerance (such as Bi, Sb, and Pb metals) for morphology control of Pt-based intermetallic compounds during thermal annealing progress. This could also be coupled with the improved molten salt technology [168, 169]. Although few classes of solution-generated Pt-based intermetallic NCs with morphology control have been reported, the success of Pt-THH development using a solid-state protocol [80] possibly pave the way for dealing with those disorder-to-order transitions at high temperatures (> 500 °C) with shape preservation.
